# A Systematic Screen to Discover and Analyze Apicoplast Proteins Identifies a Conserved and Essential Protein Import Factor

**DOI:** 10.1371/journal.ppat.1002392

**Published:** 2011-12-01

**Authors:** Lilach Sheiner, Jessica L. Demerly, Nicole Poulsen, Wandy L. Beatty, Olivier Lucas, Michael S. Behnke, Michael W. White, Boris Striepen

**Affiliations:** 1 Center for Tropical and Emerging Global Diseases, University of Georgia, Athens, Georgia, United States of America; 2 School of Chemistry and Biochemistry, Georgia Institute of Technology, Atlanta, Georgia, United States of America; 3 Department of Molecular Microbiology, Washington University School of Medicine, St. Louis, Missouri, United States of America; 4 Department of Molecular Medicine, University of South Florida, Tampa, Florida, United States of America; 5 Department of Cellular Biology, University of Georgia, Athens, Georgia, United States of America; University of Michigan, United States of America

## Abstract

Parasites of the phylum Apicomplexa cause diseases that impact global health and economy. These unicellular eukaryotes possess a relict plastid, the apicoplast, which is an essential organelle and a validated drug target. However, much of its biology remains poorly understood, in particular its elaborate compartmentalization: four membranes defining four different spaces. Only a small number of organellar proteins have been identified in particular few proteins are known for non-luminal apicoplast compartments. We hypothesized that enlarging the catalogue of apicoplast proteins will contribute toward identifying new organellar functions and expand the realm of targets beyond a limited set of characterized pathways. We developed a bioinformatic screen based on mRNA abundance over the cell cycle and on phyletic distribution. We experimentally assessed 57 genes, and of 30 successful epitope tagged candidates eleven novel apicoplast proteins were identified. Of those, seven appear to target to the lumen of the organelle, and four localize to peripheral compartments. To address their function we then developed a robust system for the construction of conditional mutants via a promoter replacement strategy. We confirm the feasibility of this system by establishing conditional mutants for two selected genes – a luminal and a peripheral apicoplast protein. The latter is particularly intriguing as it encodes a hypothetical protein that is conserved in and unique to Apicomplexan parasites and other related organisms that maintain a red algal endosymbiont. Our studies suggest that this peripheral plastid protein, PPP1, is likely localized to the periplastid compartment. Conditional disruption of PPP1 demonstrated that it is essential for parasite survival. Phenotypic analysis of this mutant is consistent with a role of the PPP1 protein in apicoplast biogenesis, specifically in import of nuclear-encoded proteins into the organelle.

## Author Summary

Apicomplexa are a group of parasites that cause important diseases, including malaria and several AIDS associated opportunistic infections. The parasites depend on an algal endosymbiont, the apicoplast, and this provides an Achilles' heel for drug development. We use *Toxoplasma gondii* as a model to characterize the biology and function of the apicoplast. In this study we apply a strategy to identify new apicoplast proteins and to prioritize them as potential targets through the analysis of genetic mutants. To aid this goal we develop a new parasite line and a protocol enabling the streamlined construction of conditional mutants. Using this new approach we discover numerous new apicoplast proteins, many of them have no assigned function yet. We demonstrate that function can be deduced using our genetic approach by establishing the essential role in apicoplast protein import for a new factor with intriguing localization and evolutionary history.

## Introduction

Apicomplexa are a phylum of single-celled eukaryotes. All members of the phylum are parasites and a number of species are responsible for important human (malaria and toxoplasmosis) and livestock (babesiosis, theileriosis and coccidiosis) diseases. Of these, malaria is the most significant global health problem with a billion people at risk of infection and millions of cases annually. Treatment of malaria is constantly threatened by the ability of the parasite to rapidly develop drug resistance. *Toxoplasma* infections are even more common but do not usually result in overt disease. However, severe toxoplasmosis occurs in immunocompromised individuals and during congenital infection. *Toxoplasma* has also proven to be a reliable model for the study of those aspects of parasite biology that are shared among the members of the phylum.

One of the current prime targets for the development of new anti-apicomplexan drugs is the apicoplast. The apicoplast is a unique chloroplast-like organelle present in most apicomplexans, including the agents of malaria and toxoplasmosis. While no longer photosynthetic, the apicoplast is a center of metabolic activity harboring several major anabolic pathways [1], [2]. The particular importance of each of these pathways varies between parasite species and specific host cell niches. Apicoplast fatty acid synthesis, for example, is essential in *T. gondii* and in the liver stage of *Plasmodium* but dispensable in the erythrocytic phase of malaria [3]–[5]. In contrast, apicoplast isoprenoid synthesis appears to be more uniformly essential and may represent the primary function of the organelle [6]–[9]. Regardless of these specific differences, numerous aspects of apicoplast metabolism and its biogenesis and maintenance are essential for the parasite, and multiple enzymes have been validated as targets genetically and/or chemically. It is likely that many more such targets are yet to be discovered.

**Figure 1:**
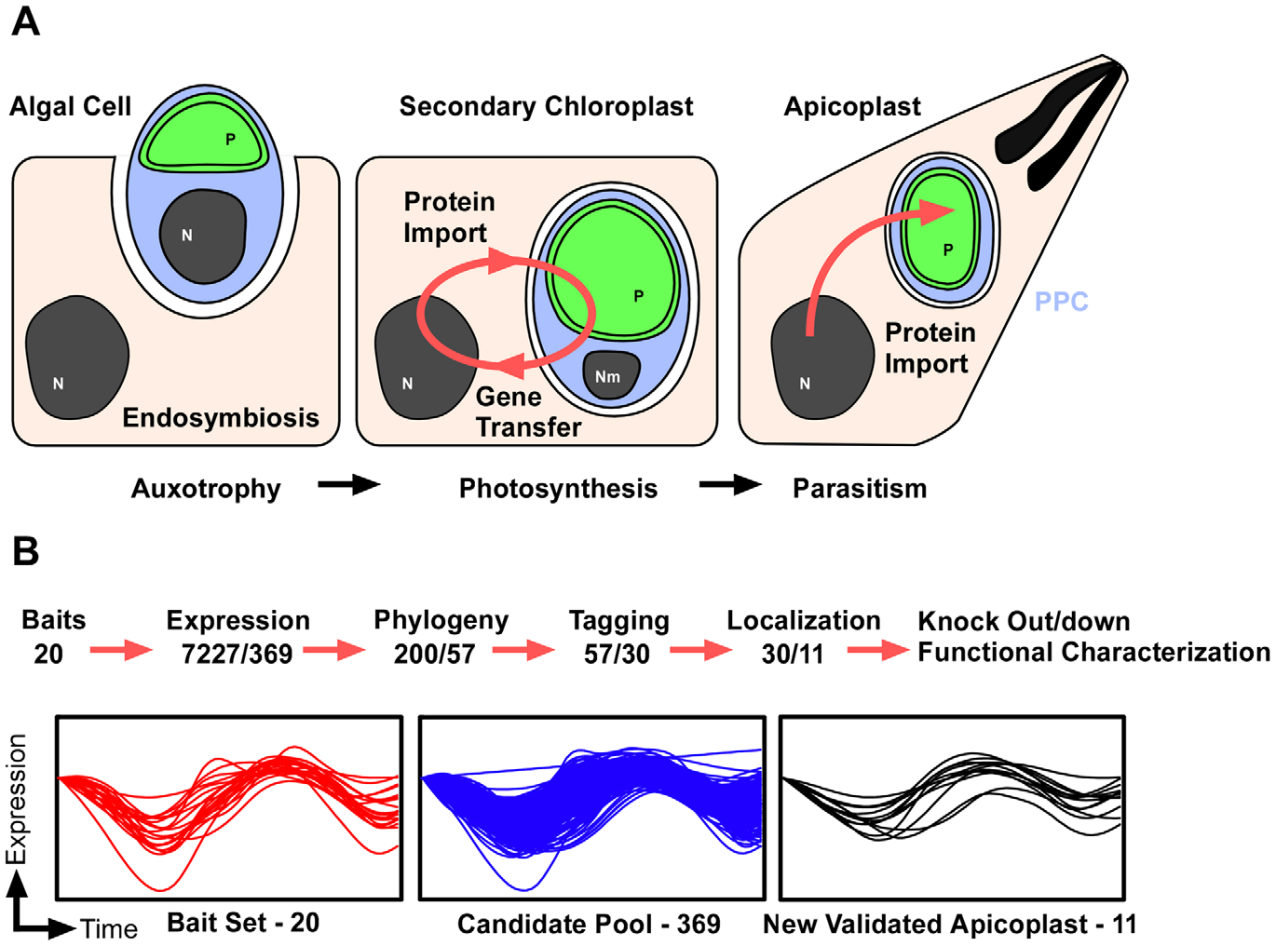
Discovering new apicoplast proteins weighing multiple data sources. (A) Schematic outline of the acquisition and evolution of the apicoplast. An algal cell (blue) carrying a chloroplast (green) enters an endosymbiotic relationship with a protist host. The establishment of a protein import system allows for massive gene transfer and the development of a stable union. The initial compartments of the endosymbionts are highly conserved over long periods of evolutionary time. PPC, the periplastid compartment is highlighted in blue. (B) Overview of the pipeline used for the identification, prioritization, confirmation and disruption of apicoplast proteins. The graphs show mRNA abundance profiles over two consecutive tachyzoite cell-cycles as described in [36]. Profiles are shown for those proteins used as seeds (left), the resulting candidate pool (middle), and for the newly identified apicoplast proteins (right).

The evolutionary history of the apicoplast is fascinatingly complex as it is derived from two independent endosymbiotic events. In the first step, endosymbiosis of a photosynthetic cyanobacterium in a eukaryotic auxotroph gave rise to primary plastids. These include the chloroplasts of plants and green and red algae. In the second step, a single-celled red alga was engulfed by a phagotrophic protist (Figure 1A). This alga was retained and eventually transformed into a fully dependent endosymbiont organelle [10]. According to the chromealveolate hypothesis, a single secondary endosymbiotic event involving a red alga lies at the origin of a very large and tremendously diverse group of organisms. The chromealveolates span all the way from single-celled predators and parasites to complex multicellular organisms like the large kelps [11]. While this hypothesis has been challenged at various points (reviewed in [12]), several independent phylogenetic analyses support a robust relationship between the apicoplast and the secondary plastids of other members of the Chromalveolata [10], [13]–[16].

Each of the two consecutive endosymbioses was accompanied by gene transfer from the genome of the endosymbiont to the host genome. As a result the vast majority of apicoplast proteins are nuclear encoded. Importantly, nuclear encoded apicoplast proteins are of varied origin: some are derived from the cyanobacterium, others are of eukaryotic origin and trace back to the algal endosymbiont or the second eukaryotic host and represent a later invention or adaptation. The complex origin of the apicoplast is not only evident in its genetic makeup but also has morphological consequences, most visible in the presence of four surrounding membranes. The two inner membranes correspond to the chloroplast membranes, which themselves are thought to be homologous to the two membranes of the cyanobacterium [17]. The second outermost membrane, often referred to as the periplastid membrane, is considered a remnant of the algal plasma membrane. Finally the outermost membrane is believed to derive from the host endomembrane system [18]–[20]. Four membranes define four compartments: The inner most is the apicoplast lumen or stroma. This is the most voluminous and home to the organellar genome and most of the apicoplast metabolic pathways characterized to date. Information on the biology of the outer compartments is limited, as very few of their proteins have been discovered as of yet.

Nuclear encoded proteins travel to the apicoplast using an elaborate and still poorly understood system of signals and import machineries. For most stromal apicoplast proteins, trafficking depends on a bipartite leader at their N terminus [21], [22]. Not all peripheral proteins carry such a signal [13], [23]–[26]. Apicoplast proteins are likely cotranslationally imported into the endoplasmic reticulum, and then travel to the apicoplast through the secretory pathway. This step is the least understood but may follow an endosomal route [27], delivering proteins to the outermost compartment of the organelle. Three protein translocons have been recently described that translocate protein cargo across the remaining apicoplast membranes. Passage through the periplastid membrane was proposed to be accomplished using an endosymbiont-derived endoplasmic reticulum-associated degradation translocon [13], [28]–[30]. Genetic evidence from *T. gondii* demonstrated that apicoplast import depends on one of the components of this complex, Der1 [13]. Transport over the two inner apicoplast membranes appears mediated by complexes homologous to the translocon of the outer and the inner chloroplast membranes (TOC/TIC) [20]. A small number of putative components of these complexes have been identified in apicomplexans, including TIC20, TIC22, and TOC75 [28], [31], [32], and their critical role in import has been validated through mutant analysis. The import machinery is an obvious candidate for intervention. It employs a large number of potential target enzymes of divergent origin, and a block on import is bound to inhibit most apicoplast functions.

We hypothesize that enlarging the catalogue of confirmed apicoplast proteins would provide a fuller understanding of apicoplast biology and ultimately lead to novel chemotherapeutic targets. To date, two major approaches have been used to identify apicoplast proteins. One is based on the identification of homologs of specific pathways or proteins previously described in the plastids of other organisms. Driven by the complete genome coverage of a large number of Apicomplexa, this has been very successful [1]. The second approach uses bioinformatics to search for bipartite targeting motifs in the N-terminus of proteins encoded in the *P. falciparum* genome [33]. Proteins that lack either the targeting signal or known plastid homologs remain undetected by both approaches. Here, we extended these efforts by taking advantage of functional genomic data to discover novel apicoplast proteins that may not be readily identifiable. We then prioritized candidates based on the phyletic distribution of their homologs. We experimentally tested a first set of genes in *T. gondii* (Figure 1B). From 30 localized proteins we report the identification of 11 new apicoplast proteins found within different sub-compartments of the organelle. To address their function, we developed a robust system for rapid gene disruption, and demonstrate its efficiency by generating mutants for two essential genes, encoding one luminal and one peripheral plastid proteins.

## Results

### Identification of candidate apicoplast proteins based on their gene expression profile

To more fully understand the many functions of the apicoplast, we wished to establish a comprehensive catalog of organellar proteins. To this end, we developed a multi-step pipeline to prioritize potential candidates. In the first step, we paid particular attention to the timing of gene expression. Behnke and colleagues have recently discovered common periodic profiles of mRNA abundance for distinct organellar proteins in *Toxoplasma*. This cell cycle timing appears to coincide with the formation of the organelles. Similar time-ordered relationships leading to shared transcription patterns had previously been observed for organellar biogenesis during replication of *Plasmodium* merozoites in the red blood cell [34]. Thus, co-regulation of transcription might be used as one criterion to identify unknown components of organelles [35], [36]. To explore whether timing of mRNA expression could be used to identify new apicoplast genes, we compiled a list of 20 proteins, for which apicoplast localization had been confirmed in *T. gondii* and *Plasmodium* (Table S1). The mRNAs encoding these proteins have similar cell cycle profiles with peak levels during the G1 to S phase transition [36]. A group of 369 candidate genes (Figure 1B, Table S2) matched this specific periodic pattern with high correlation (GeneSpring 11.0, Pearson correlation at 0.05 FDR). In addition to the original 20 apicoplast proteins the expanded list captured at least 8 previously confirmed apicoplast proteins and proteins from secondary plastids of other organisms (Table S1) demonstrating the predictive value of this approach. To assess the efficiency of this filter, we selected two sets of control genes. One set contained 200 genes whose periodic pattern is inverse to the median expression profile of the 369 genes (R3 and R4 in [36]), none of the confirmed apicoplast genes was found here. Additionally, 200 genes were selected at random from ToxoDB in 3 independent experiments, those included 0, 0 and 2 of the confirmed apicoplast proteins. This suggested significant enrichment in the list used.

In order to further assess candidates and to derive a more focused list suitable for experimental validation, we applied a secondary screen based on molecular phylogeny. The rationale behind this second filter is the hypothesis that genes encoding apicoplast proteins follow a phyletic distribution that is consistent with the evolutionary path that led to the acquisition of the organelle (Figure 1A). The predicted protein sequences of the first 200 candidates in the expression list were retrieved from the *T. gondii* genome database (http://toxodb.org/toxo/) and used to perform BLAST searches against the NCBI nucleotide collection database (http://blast.ncbi.nlm.nih.gov/), and the results were systematically analyzed. A set of rules was developed by which we scored the presence or absence of homologs in different clades, and the degree of statistical confidence associated with that identification. Based on these rules we assigned positive or negative points to candidates (see Materials and Methods for details). The parameters were iteratively adjusted such that a positive score (1 or more) was obtained for all the 28 known apicoplast proteins (Table S1) mentioned above. Accordingly only genes with positive scores were considered candidates for further characterization. Half (49.5%) of the genes analyzed met this second requirement, and the first 57 genes (Table S3) were selected as a manageable number to further test experimentally.

### Identification of 11 novel apicoplast proteins

The next step in our pipeline was to determine experimentally whether a candidate gene product indeed targets to the plastid. We introduced a DNA cassette encoding an epitope-tag and a selectable marker directly into the native gene locus via homologous recombination. The efficiency of in-locus tagging is dramatically increased in *T. gondii* mutants that lack *TgKu80*, a key component of the non-homologous end-joining repair machinery [37], [38]. In this mutant, stable transformation occurs predominantly through homologous recombination. Using the approach described by Huynh and coworkers we attempted 3' endogenous tagging for each of the 57 candidates.

**Figure 2:**
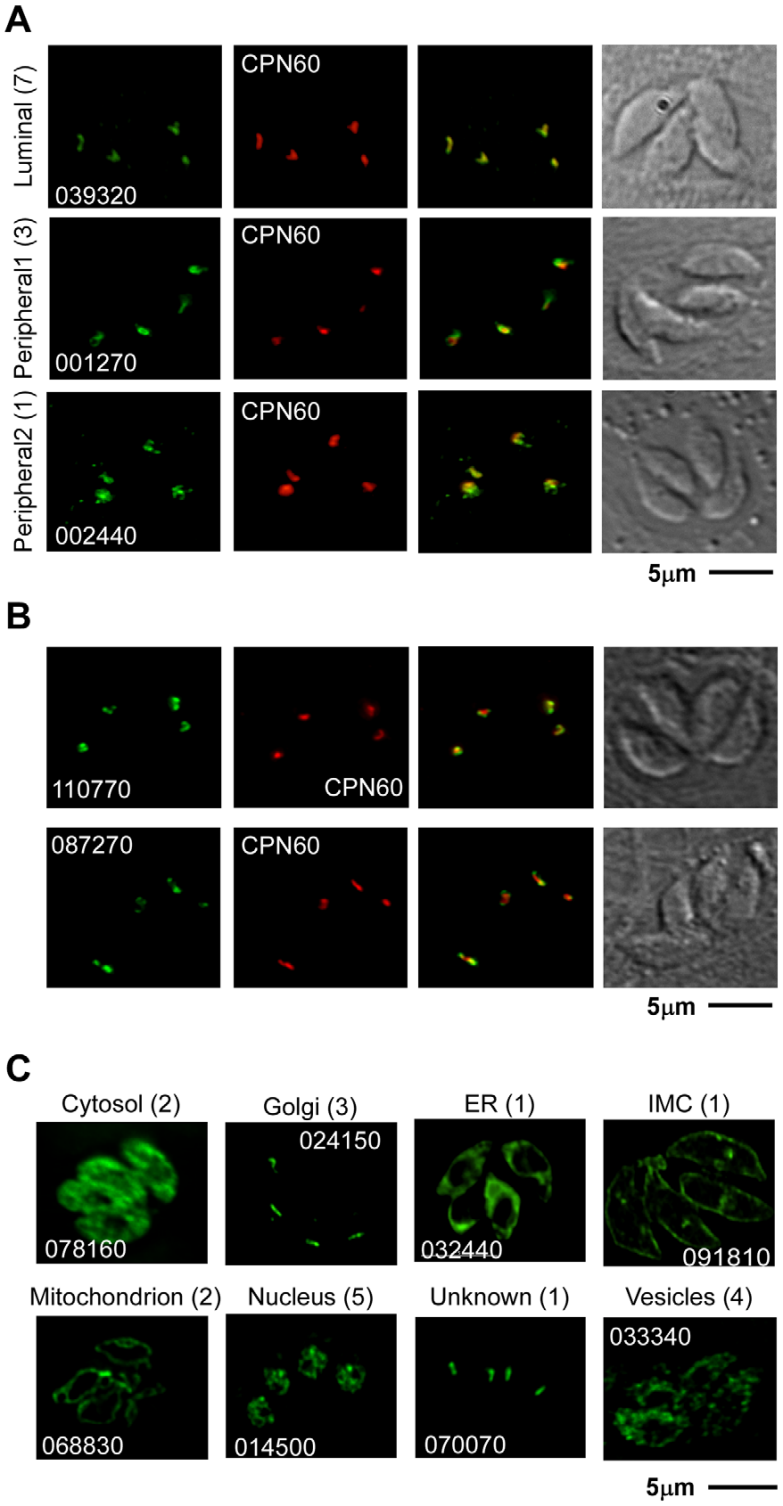
Localization of 30 previously uncharacterized proteins by endogenous epitope tagging. Fluorescence microscopy analysis of parasites expressing endogenously HA-tagged proteins. (A) shows examples of a luminal apicoplast protein (top panel), a protein localizing to the periphery of the apicoplast (middle panel), and an example for the second patchy peripheral pattern observed (bottom panel). Numbers on the left represent the numbers of proteins showing the described pattern. (B) shows representative image of ATrx2 (top panel) and of PPP1 (bottom panel) localizations. (C) Representative images of tags that resulted in the identification of non-apicoplast proteins. Likely target organelles are indicated. Scale bar = 5 µm. HA in green, CPN60 in red, numbers reflect the ToxoDB gene ID as detailed in the Results section.

After manually reassessing the predicted gene models (see Materials and Methods) we designed primers to generate targeting plasmids through ligation independent cloning for all 57 genes (Table S3). Sequences for 48 genes were successfully amplified and cloned into p3HA.LIC.CATΔpac (see Materials and Methods). Each of the resulting vectors was linearized using a unique restriction site contained within the gene specific sequence (Table S3). DNA was transfected into the newly engineered TATiΔKu80 line (see below for details) and chloramphenicol resistant parasites were established with all 48 constructs. In 30 of these polyclonal pools of stably transfected parasites, we detected unambiguous expression of the epitope-tag by immunofluorescence assay (IFA) (Table S3). 11 candidates (Table 1) were localized to the apicoplast or its immediate proximity (Figure 2A,B and Figure S1). Other localizations observed included mitochondrion (2 gene products), nucleus (5), Golgi apparatus (3), cytosol (2), endoplasmic reticulum (1), vesicular pattern (4) and unknown compartments (2) (Table S3, Figure 2C and Figure S2). Apicoplast-localized gene products therefore make up 36% of the total candidates localized in this study.

**Table 1:** Summary of the bioinformatics and experimental data for each of the eleven new apicoplast proteins found in this work.

Gene ID	predicted product	predicted SP/TMD	predicted Domains	homology	Pf homologue	PlasmoAP	Sub localization	Cosmid	result of cosmid attempt
TGME49_110770	hypothetical protein, conserved	SP, TMD* (TMHMM AA9–31)	0044270 Thioredoxin-like	algae and apicomplexans	PFE1450c	no	periphery	PSBLW05	resistance, no KO
TGME49_021920	hypothetical protein	none	NifU NifU-like domain, NIFU NIFU NITROGEN-FIXING C-TERMINAL FIXATION NITROGEN NIFU-LIKE DOMAIN CLUSTER HESB/YADR/YFHF:NITROGEN-FIXING	plants	none	-	lumen	PSBMB21	KO (not essential)
TGME49_021330	DNA gyrase subunit A, putative	none	0035588 Type II DNA topoisomerase, DNA gyrase A, more	cyanobacteria, algae	PFL1120c	yes	lumen	NA	
TGME49_008840	ATP-dependent DNA helicase, putative	none	0044179 Nucleic acid-binding proteins, 0044263 P-loop containing nucleoside triphosphate hydrolases, ultradead3, HELICASE_ATP_BIND_1 Superfamilies 1 and 2 helicase ATP-binding type-1 domain profile, more		none	-	lumen	PSBM109	KO (not essential)
TGME49_087270	hypothetical protein	TMD (TMHMM 87-109)	none	algae and apicomplexans	PFL0600w	yes	periphery	ToxoW30	resistance, no KO, essential
TGME49_039680	hypothetical protein, conserved	none	none	apicomplexan unique/specific	PFE1075c	yes	lumen	PSBMG78, PSBLE72	resistance, no KO
TGME49_091670	RNA helicase, putative	SP	0044263 P-loop containing nucleoside triphosphate hydrolases, ultradead3, DEAD DEAD/DEAH box helicase, HELICASE_ATP_BIND_1 Superfamilies 1 and 2 helicase ATP-binding type-1 domain profile, more	only Tg, plants and algae, few mammalians	PF10_0209	no	lumen	NA	
TGME49_059230	hypothetical protein	SP, TMD* (TMHMM AA20-42)	0045587 lambda integrase-like, N-terminal domain, SAP SAP motif profile	apicomplexan unique/specific	MAL13P1.42	yes	lumen	NA	
TGME49_039320	hypothetical protein, conserved	TMD (TMHMM AA54-76)	0041621 BolA-like, BolA BolA-like protei	red algae, plants	no	-	lumen	NA	
TGME49_002440	hypothetical protein	SP	none	only Tg, Nc, Phaeodactylum tricornutum	no	-	in and near or around the apicoplast	NA	
TGME49_001270	hypothetical protein	none	none	Only Tg, Nc, Theileria	PF10_0246	yes	periphery	TOXOZ18, TOXPD77, TOXPI78	cosmid sequence rearranged

Note that some of the non-apicoplast localized proteins, may nevertheless be involved in apicoplast biogenesis. Such may be the case for vesicular proteins. In that context TGME49_070070 is of particular interest. The predicted protein encoded by this gene contains a C-terminal transmembrane domain and share several domains with proteins known for their role in vesicle trafficking. The protein does not overlap with markers of the Golgi apparatus or the apicoplast (Figure S2), and its precise subcellular localization is yet to be determined.

### Six new luminal apicoplast proteins have likely roles in genome maintenance and metabolism

As documented by IFA, the 11 new apicoplast proteins showed variation in their staining pattern, suggesting that they might target to different sub-compartments within the apicoplast. We divided them into two initial groups: the first group consists of proteins that fully co-localize with the luminal apicoplast marker CPN60 [13] indicating that they likely are also luminal proteins. The second group consists of proteins that, while consistently in proximity, have little or no direct overlap with staining from CPN60 (Figure 2A,B Figure S1, Table 1).

To gain insight into the function of these proteins we subjected their amino acid sequences to a variety of bioinformatic analyses (Table 1). We found three to be predicted membrane proteins (110770, 059230 and 039320; we refer here to genes and proteins by the number component of the corresponding gene ID in ToxoDB, so that TGME49_110770 becomes *110770*). We then identified several conserved functional domains (Table 1). The first of those is encoded by *021330*, a homolog of the subunit A of the bacterial type II topoisomerase, DNA-gyrase. The genomes of *T. gondii* and of the *Plasmodium spp*. each encode two gyrase subunits (GyrA and GyrB), and both were studied in *Plasmodium* [39], [40]. While the apicoplast targeting of 021330 was not surprising based on its *Plasmodium* homolog, other proteins found in the luminal group were less readily anticipated. Three of those are also likely involved in plastid genome maintenance: *00884* encodes a presumptive ATP-dependent DNA helicase that shows significant sequence similarity with the RecG protein of Gram-negative bacteria. RecG is a double-stranded DNA translocase that is involved in DNA recombination and repair [41]. *059230* encodes a domain found in phage-integrases and DNA break-rejoining enzymes, suggesting that this protein is also part of the machinery that mediates apicoplast DNA recombination events. The third protein, 091670, contains domains typically found in RNA-helicases.

The luminal group also includes *039320*, which encodes a protein with a Bol-A like domain at its C-terminus. The exact molecular function of BolA proteins is still under study. *Escherichia coli* BolA is suggested to be implicated in the tolerance towards different environmental pressures, since its expression is induced under stress conditions. This leads to the reduction of cell surface area, via switching between elongation and septation systems during cell division [42]. While these observations propose a potential role in transcription regulation, a recent study raises a second interesting possibility in the context of plastids. A component of the *Arabidopsis thaliana* plastid Iron-Sulfur cluster pathway, SufE, was reported that also contain a BolA domain in its C-terminus [43]. A second protein found in our screen is proposed to be involved in the same pathway: whereas the product of *021920* is annotated as a hypothetical protein, more careful recent analysis suggests its involvement in iron-sulfur cluster biogenesis as a NifU-like scaffold protein [2].

Taken together proteins from the first group show predicted domains that tie them to luminal functions of the plastid consistent with their observed localization. The last luminal apicoplast protein, 039680 (Figure S1), has no predicted domain from which a putative function could be inferred.

### Four new proteins with non-luminal apicoplast localizations

**Figure 3:**
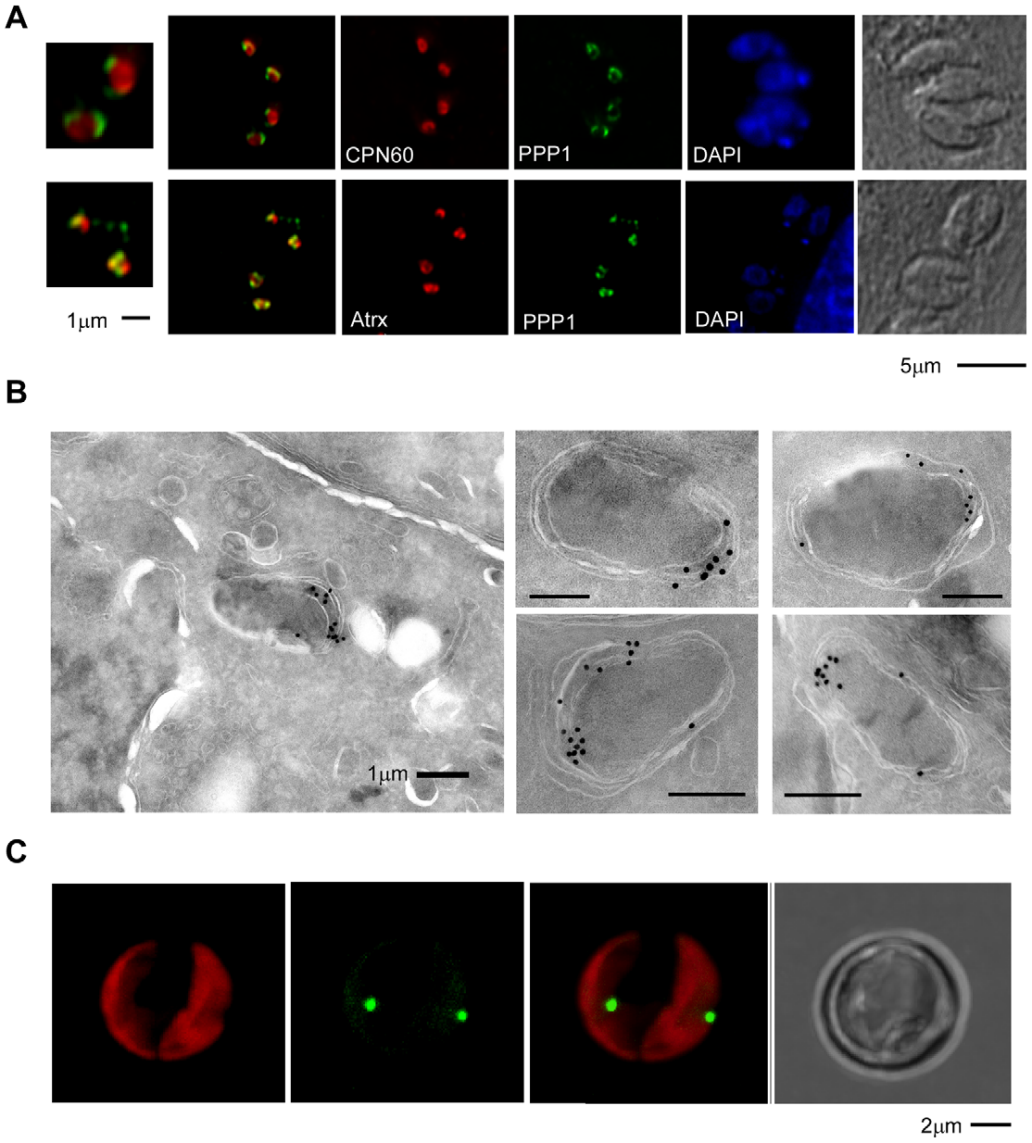
PPP1 is a resident of the apicoplast periphery and most likely the periplastid compartment. (A) Fluorescence microscopy analysis of parasites expressing endogenously HA-tagged PPP1 (in green in both panels), co-stained with the luminal marker CPN60 (red in upper panel) and peripheral marker ATrx1 (red in lower panel). Note that in both cases the signals do not overlap completely. Scale bar = 5 µM, and scale bar for magnified insert = 1 µM. (B) Electronmicrographs of parasites expressing endogenously HA-tagged PPP1 using immuno-gold staining after cryo-sectioning. The images on the right show only the apicoplast. Scale bar - 1 µm. (C) *T. pseudonana* cells were transfected with a 267353–GFP encoding plasmid and stable transgenics were established by drug selection. Expression of the transgene was induced by nitrate. Note GFP fluorescence (green) at the center of the chloroplast (imaging chlorophyll autofluorescence in red). Scale bar 2 µm.

The staining pattern of four of the new apicoplast proteins suggested a potential non-luminal localization within the apicoplast. The protein encoded by *002440* is found in close proximity to CPN60 but shows additional punctate staining around the plastid that has not been previously reported (Figure 2A). The product of *001270* shows staining that is very similar to the previously described circum-plastid localization of ATrx [23] (Figure 2A). Finally the proteins encoded by *110770* (Figure 2B) and *087270* (Figures 2B, 3A) show a patchy peripheral apicoplast localization reminiscent of the localization of Der1, UfD1 and CDC48 proteins that are believed to reside in the periplastid compartment (PPC) – a compartment equivalent to the cytoplasm of the algal endosymbiont [13]. To follow this further we performed cryo-electron microscopy using the endogenously HA-tagged 087270 cell-line, as this protein appeared to be expressed at the highest level. Cryosections were stained with HA specific antibodies followed by gold labeled anti-immunoglobulin. We observed strong labeling of the periphery of the apicoplast, while gold beads were absent from the lumen of the organelle (Figure 3B). Interestingly, we noticed a polarized distribution of the protein in the apicoplast periphery.

### Two proteins conserved in and unique to the red lineage

Studying the two presumptive PPC proteins in greater detail we noticed a restricted distribution across the tree of life: tBLASTn searches against GenBank and various additional nucleotide databases (including recent EST, GSS and environmental sampling efforts) revealed homologs of these genes only in a relatively small collection of organisms. Specifically, these proteins are restricted to chromalveolates that maintain a plastid, and to red algae. We further noted that the homologs found in the cryptomonads *Guillardia theta* and *Hemiselmis andersenii* are encoded in the nucleomorph, the relict nucleus of the algal symbiont that resides in the PPC of some secondary plastids [44]. Both these nucleomorphs are of red algal origin, while the nucleomorph of the chlorarachniophyte *Bigelowiella natans* is of green algal origin, and does not encode a homolog of this protein.

**Figure 4:**
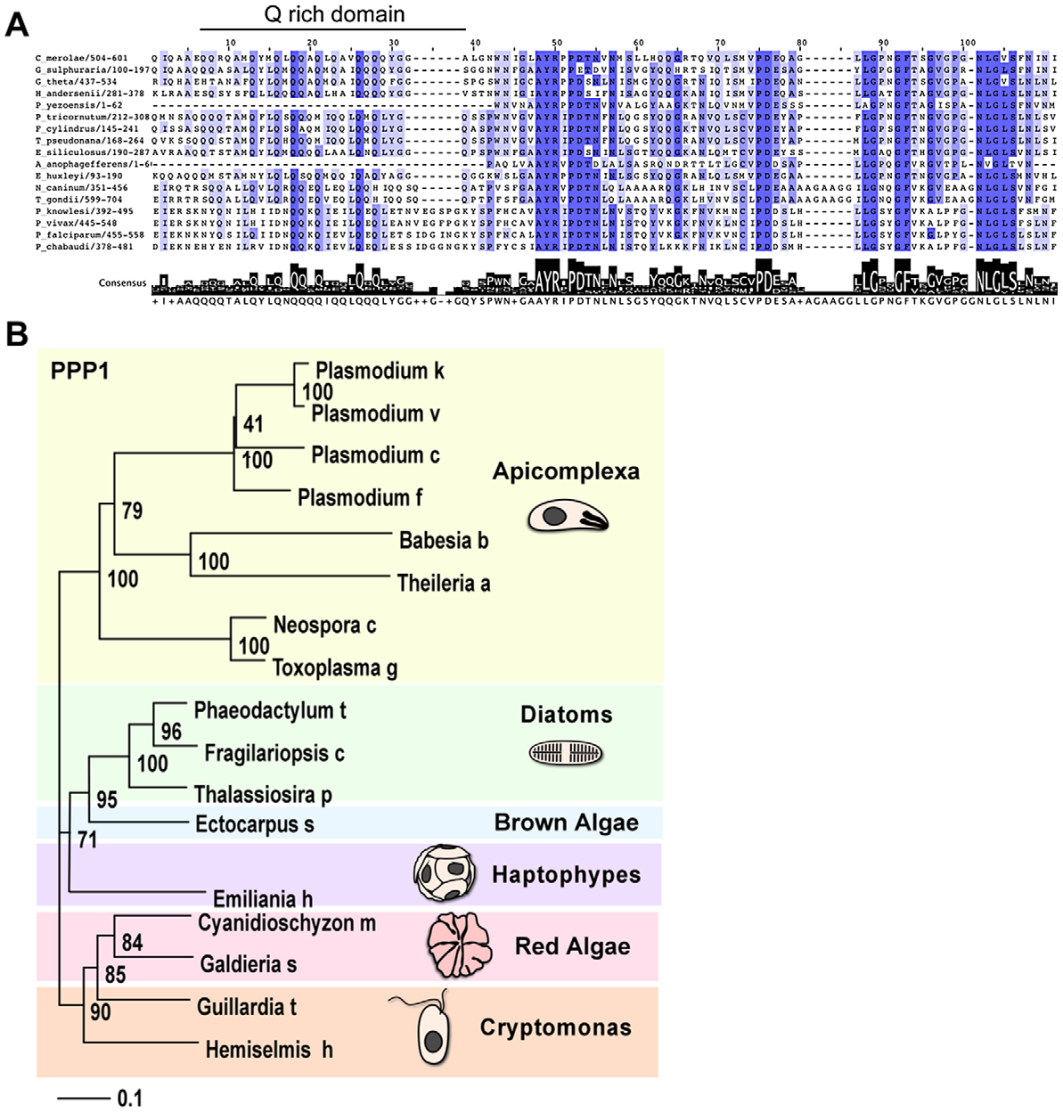
PPP1 is conserved specifically among members of the red algal lineage. (A) Multiple protein alignment of the putative orthologues of PPP1. Color gradient corresponds to sequence similarity, deep blue represents 100% identity. Size of black bars indicates the level of conservation across the consensus. (B) Neighbor joining tree of PPP1. Bootstrap values are indicated for each node.

We performed multiple sequence alignments of the putative orthologs of each of the two putative *T. gondii* PPC proteins. In both cases we observed a core region conserved among all sequences (Figure 4A and Figure S5B). For 110770, this conserved region corresponded to a predicted thioredoxine-like domain (Trx, superfamily 52833, Figure S5B). The statistical support for a Trx-like domain is not equally strong in all homologs (the E-value for *the T. gondii* protein is 4e–03). However, we note the invariant presence of a C-X-X-C motif, the critical signature of Trx-domains [45], in all sequences (Figure S5B). These observations suggest 110770 to be a second plastid-resident thioredoxin-like protein in *T. gondii*, which we here name ATrx2.

For 087270 the high similarity block was found at the carboxy-terminal end and revealed a conserved glutamine (Q) rich patch (Figure 4A). We subjected both protein families to phylogentic analyses (see Materials and Methods). Figure 4B shows a neighbor-joining tree derived from 140 unambiguously aligned residues of 087270. The apicomplexan proteins form a well-supported clade, diatoms and brown algae form a sister clade to the apicomplexans, and cryptomonads and red algae are the members most distant to apicomplexans.

We note that while electron microscopy supported localization of 087270 to the apicoplast periphery, its resolution is generally not sufficient to distinguish between the various peripheral compartments. As described previously, the distances between these compartments are similar to the size of the antibodies used for labeling [13], and this makes a firm assignment of a precise subcompartment in apicomplexans difficult. However, in diatoms, periplastid proteins appear to localize to a distinct and identifiable compartment referred to as the blob-like structure at the center of the usually bilobed chloroplast [46]. We identified a diatom homolog for 087270, and decided to localize it in *Thalassiosira pseudonana*, a genetically tractable diatom. The coding sequence of the corresponding gene (protein ID 267353, JGI: Joint Genome Institute: www.jgi.doe.gov/)) was amplified from cDNA and introduced into a diatom expression vector under the nitrate reductase (NR) promoter, resulting in a C-terminal GFP fusion [47]. The resulting construct was then used to transform *T. pseudonana* by microparticle bombardment. Stable transformants were selected in the presence of CloneNAT and cloned on agar plates. Clones were propagated under nitrogen starvation and were tested for expression upon induction by growth in nitrate medium for 24 h. As shown in Figure 3C we detected GFP fluorescence mainly in discrete spots at the center of the chloroplast just outside of the plastid lumen (as detected by the red autofluorescence of chlorophyll). This localization pattern is consistent with the previously described “blob-like” structure in diatoms [46], [48]. Taken together our analyses suggest that 087270 likely resides in the apicoplast PPC, we will subsequently refer to this protein as peripheral plastid protein 1 (PPP1).

### A robust system for the isolation of conditional mutants

We next wanted to define the biological role of PPP1 using a reverse-genetic loss-of-function approach. We first attempted a direct gene knock-out. We engineered a targeting cosmid by recombineering [6] (Figure S3), replacing the coding sequence within *TOXOW30* with a chloramphenicol acetyl transferase marker. The modified cosmid was transfected into a TgKu80 knock-out background (generously shared by Huynh and Carruthers) [38]. Whereas stable drug resistant parasites were established from each of 4 independent transfections, the PPP1 locus was not disrupted in any of the 43 clones that were screened by PCR (Figure S3). These observations suggested that PPP1 may be indispensable for parasite survival. Similarly, we targeted the loci of four additional newly identified apicoplast proteins, two of which we could disrupt (Table 1 and discussion). We therefore next elected to construct conditional mutants. Several approaches have been developed to isolate conditional mutants [49]-[53]. Out of these the tetracycline-regulated transactivator system has been well suited for our studies of apicoplast biology [3], [6], [9], [13], [32]. We reasoned that we could further streamline this approach by engineering a parasite strain that combines high efficiency of homologous recombination with regulated gene expression.

**Figure 5:**
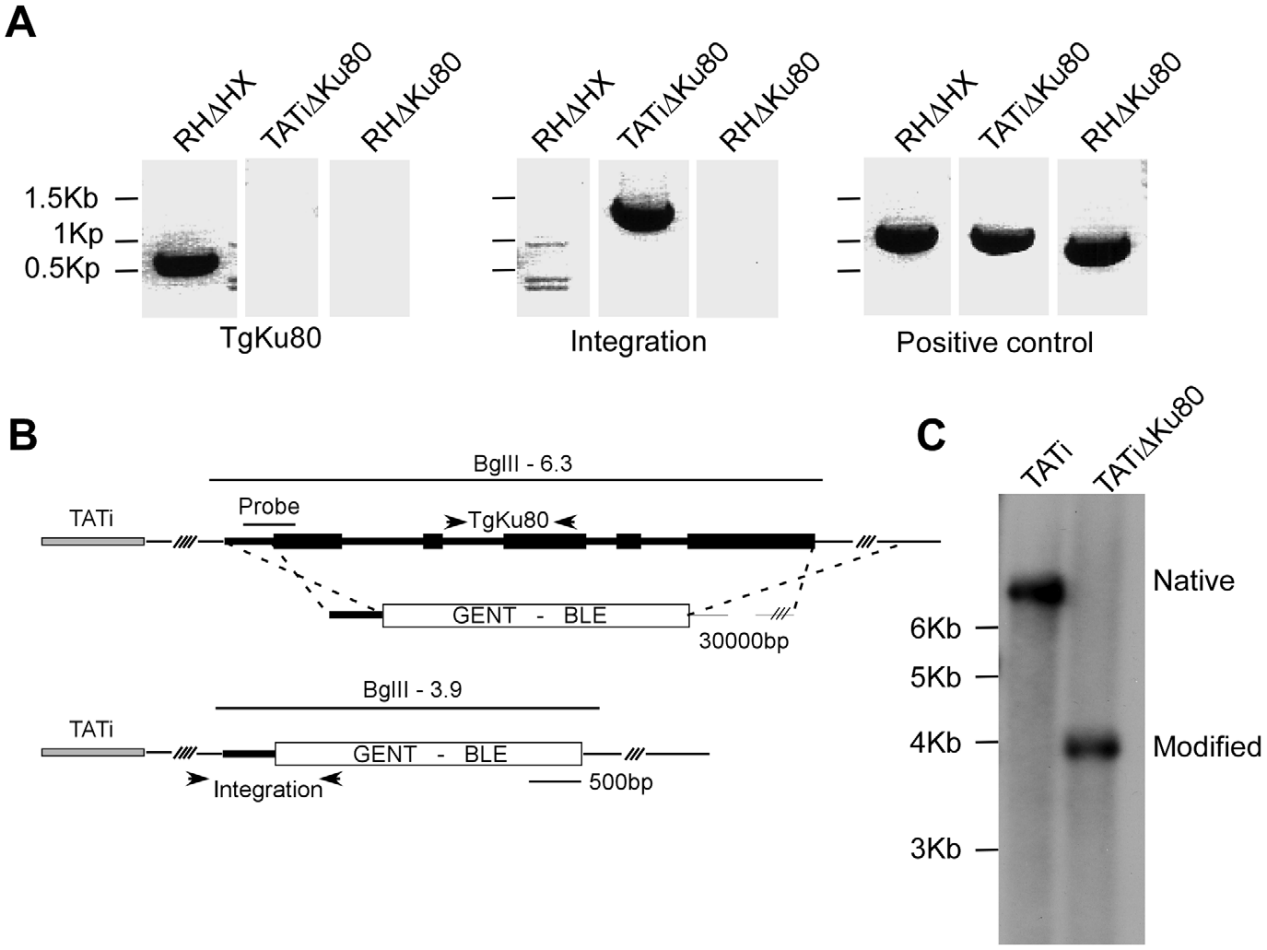
Deletion of the *TgKu80* gene in TATi transactivator parasite line. (A) PCR analysis using primers specific for the *TgKu80* coding region (left), targeting construct integration (middle), or an unrelated control region (right) using genomic DNA from RHΔHX, TATIΔ*TgKu80* and RHΔ*TgKu80* [38] as template. Position of primers is indicated in panel B. (B) Schematic representations of homologous recombination between the BLE modified *TOXOW30* cosmid and the *TgKu80* locus. The probe used for the southern blot presented in panel C is indicated and sizes expected after BglII digest are shown. (C) Southern blot analysis of the TATi*ΔTgKu80* clone and its parental TATi line demonstrating *TgKu80* disruption.

We recombineered cosmid *TOXOY50* to replace the coding sequence of the *TgKu80* locus with the phleomycin resistance marker BLE [6]. The resulting modified cosmid was then transfected into a line expressing a tetracycline-regulated transactivator (TATi) [51] and stable clones were established by phleomycin selection. A TgKu80 deletion clone was identified by PCR (Figure 5A) and successful deletion of the gene was further verified by Southern blot (Figure 5C).

**Figure 6:**
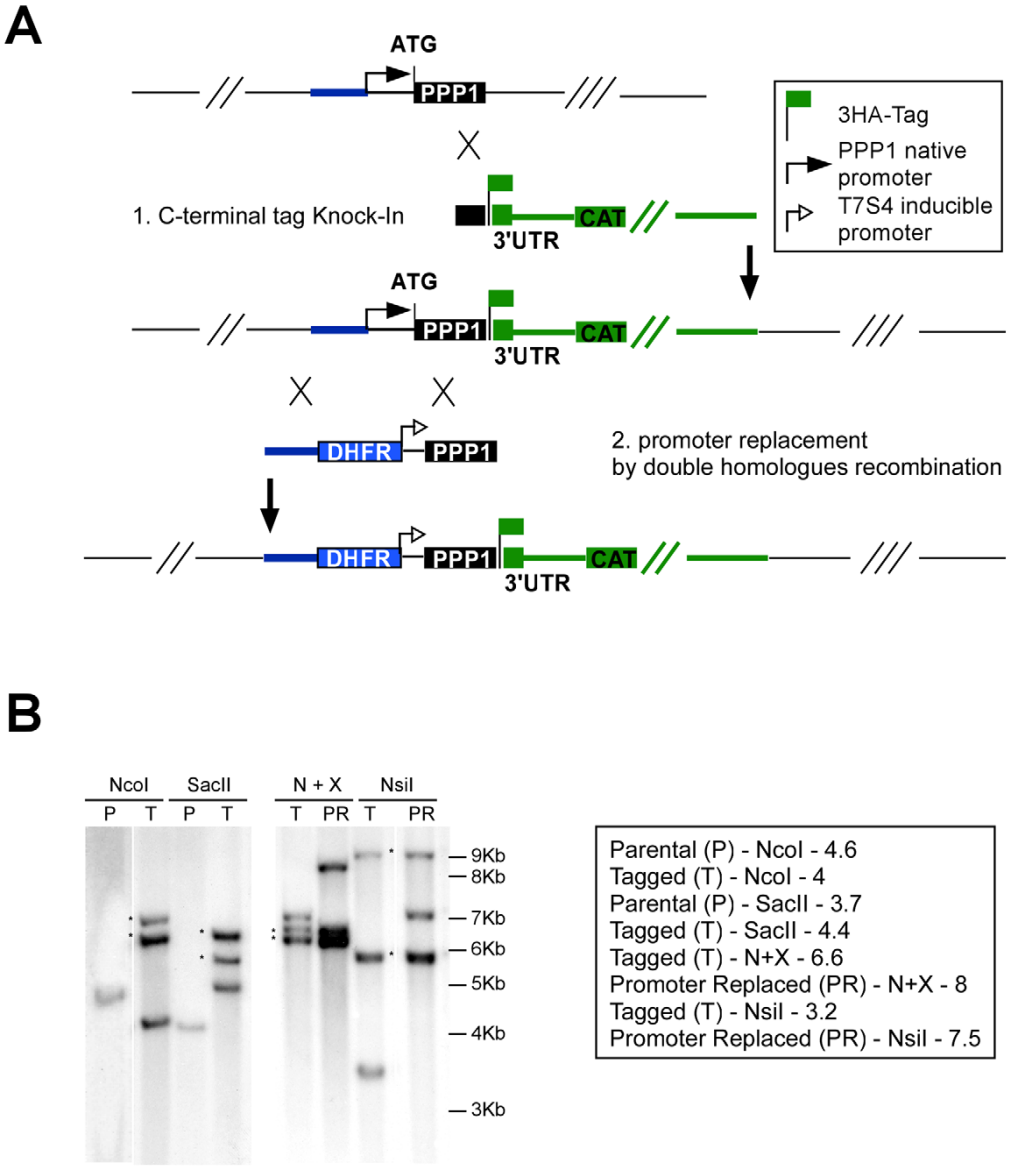
Isolation of a conditional *PPP1* mutant by promoter replacement. (A) Schematic representation of the two-step manipulation of the *PPP1* locus: First the gene is endogenously tagged by single cross-over (cassette in green), followed by promoter replacement by double-homologous recombination (cassette in blue). A legend for the tag and promoters used is show in the upper right corner. (B) Southern blot analysis before and after tagging and promoter replacement. Strains, restriction enzymes and expected band-sizes are indicated to the right. Asterisks indicate bands resulting from additional non-homologous integration of the pLIC_3HA plasmid.

In this background we tested the efficiency of an endogenous promoter replacement strategy. In this strategy, the native promoter is replaced with the tetracycline-regulated promoter [51] via double homologous recombination, resulting in a conditional knock-down mutant in a single step (see scheme in Figure 6A).

We assessed *PPP1*'s gene model experimentally and identified its true start site (at position 1,435,362 on chromosome TGME49_chrV). We then engineered a targeting plasmid that flanked a pyrimethamine resistance marker and the regulated promoter with sequences from the *PPP1* locus to replace the native promoter with this cassette (see Materials and Methods, and Figure 6A). The resulting vector was linearized with AvrII and transfected into a TATiΔKu80 background, where PPP1 was already endogenously tagged at the C-terminus as described above (Figure 6A). Clones were isolated after pyrimethamine selection and tested for promoter replacement by PCR. Both the C-terminal tagging and the promoter replacement in this (Tet)PPP1(HA) line were confirmed by Southern blot (Figure 6B, and Figure S4).

**Figure 7:**
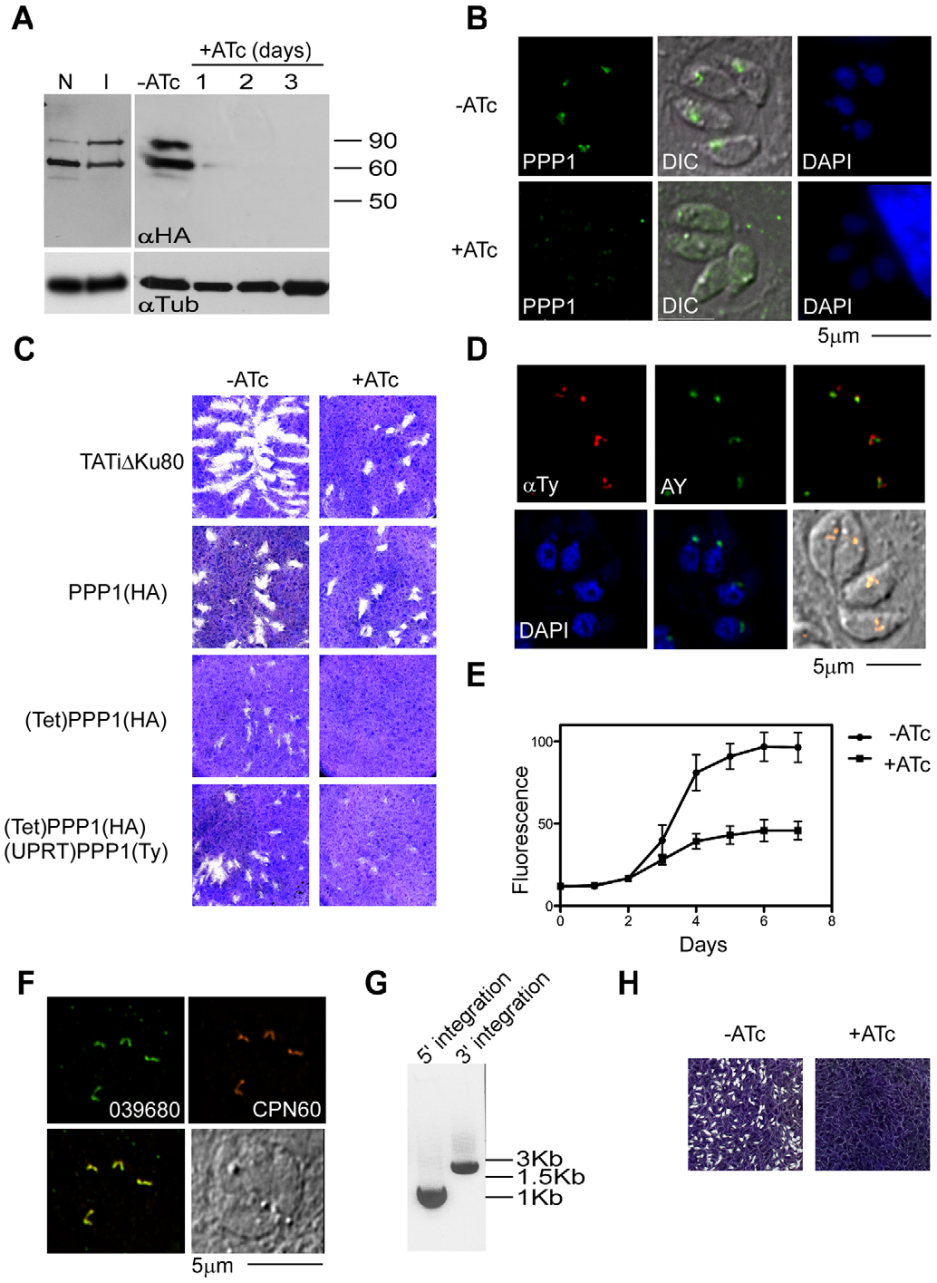
*PPP1* is essential for parasites growth. (A) Western blot analysis was of proteins extracts from PPP1(HA) and (Tet)PPP1(HA) using anti HA antibody. Note comparable levels of PPP1 when expressed from the native promoter (N) or the inducible T7S4 promoter (I). Protein levels decline swiftly upon ATc treatment, numbers indicate days of culture in 0.5 µg/ml ATc. Tubulin (lower panel) serves as a loading control. (B) Fluorescence microscopy analysis of (Tet)PPP1(HA parasites using anti-HA antibody grown in the presence and absence of ATc. (C) Plaque assays performed in the absence (-) or presence (+) of ATc. Parasite line name is indicated to the left. (D) Fluorescence microscopy of parasites expressing a second copy of PPP1, which is labeled using an anti-Ty antibody (red). The apicoplast marker used is the endogenously tagged ACP-YFP (AY, green). Scale bar = 5 µm. (E) Fluorescence growth assays show growth deficiency in ATc treated (Tet)PPP1(HA). Circles, -ATc; squares, +ATc. Each data point represents the mean of six wells, and the error bar gives the standard deviation. (F) Fluorescence microscopic analysis of endogenously tagged *039680* using anti-HA (green) and the luminal marker CPN60 (red) show that 039680 is likely a luminal apicoplast protein. (G) PCR analysis of the promoter replacement clone of *039680* with diagnostic primer sets is consistent with locus modification. The size of amplicons matches the prediction for integration at both the 5' and the 3' insertion site (900 and 1800 bp respectively). (H) Plaque assays performed with the promoter replaced *039680* cell line in the presence or absence ATc show that this gene is required for growth.

We next tested whether the inserted promoter can be used to control PPP1 expression by addition of anhydrous tetracycline (ATc) to the growth medium. We measured PPP1 levels using the endogenous HA tag by Western blot and IFA (Figure 7A,B). In the absence of ATc, we detected the two forms of the protein at a level comparable to the tagged parental strain in which expression is driven by the native promoter (Figure 7A). ATc addition resulted in marked reduction of expression, and 48 hours after addition of ATc the protein was no longer detectable (Figure 7A).

### *PPP1* is essential for parasite survival and apicoplast biogenesis

In order to establish whether PPP1 is essential for *T. gondii* survival, parasite growth was examined by plaque assay. (Tet)PPP1(HA) parasites, their parental ((PPP1(HA)) and grand-parental (TATiΔKu80) lines were all capable of forming plaques when grown in normal medium (Figure 7C). However, in the presence of ATc, no plaques were observed in the (Tet)PPP1(HA) line after incubation times as long as 12 days (Figure 7C), while both parental lines form plaques readily under these conditions. To confirm that this growth defect is solely due to depletion of the targeted gene, we re-introduced PPP1 as a Ty tagged minigene under the control of a constitutive promoter. We constructed a vector to target and disrupt the uracil phosphoribosyl-transferase (UPRT) locus and used 5-fluorodeoxyuridine (FUDR) to isolate stable transgenic clones. Integration of PPP1Ty was confirmed by PCR (not shown), as well as by IFA using antiTy antibody (Figure 7D). Complementation of (Tet)PPP1(HA) with PPP1Ty rescued the growth phenotype observed by plaque assay upon ATc treatment (Figure 7C). To time the onset of growth inhibition, an RFP-RFP transgene [32] was introduced into the (Tet)PPP1(HA) line and a clone was isolated by cell sorting [3]. We measured the growth of parasites in the presence and absence of ATc using a real-time fluorescence assay [54]. Parasites cultured in the presence of ATc showed a pronounced growth defect compared with parasites grown without ATc, and this defect became detectable on day 3 (Figure 7E). Taken together these observations demonstrate that PPP1 is essential for parasite growth, and that tightly regulated mutants can be constructed in the TATiΔKu80 strain.

To test the reproducibility of the TATiΔKu80 approach, we next attempted a promoter replacement for *039680*, one of the new luminal plastid proteins (Figure S1 and Figure 7F) that we identified (Table 1). This is a protein of unknown function, yet is conserved among apicomplexans. After testing the gene model and identifying its start site (at position 831330 on chromosome TGME49_chrVI), we targeted the promoter as described above for PPP1, and the corresponding (Tet)039680 clones were isolated after selection with pyrimethamine. Integration of the construct into the locus, and promoter replacement, were confirmed by PCR (Figure 7G). We tested the effect of ATc on the growth of these parasites by plaque assay. (Tet)039680 parasites formed plaques in the absence of ATc, but no plaques were observed upon growth in ATc (Figure 7H). We conclude that 039680 has a critical role in the lumen of the apicoplast, and further that the TATiΔKu80 provides a reliable background for the rapid construction of conditional mutants.

### *PPP1* is required for import into the apicoplast

**Figure 8:**
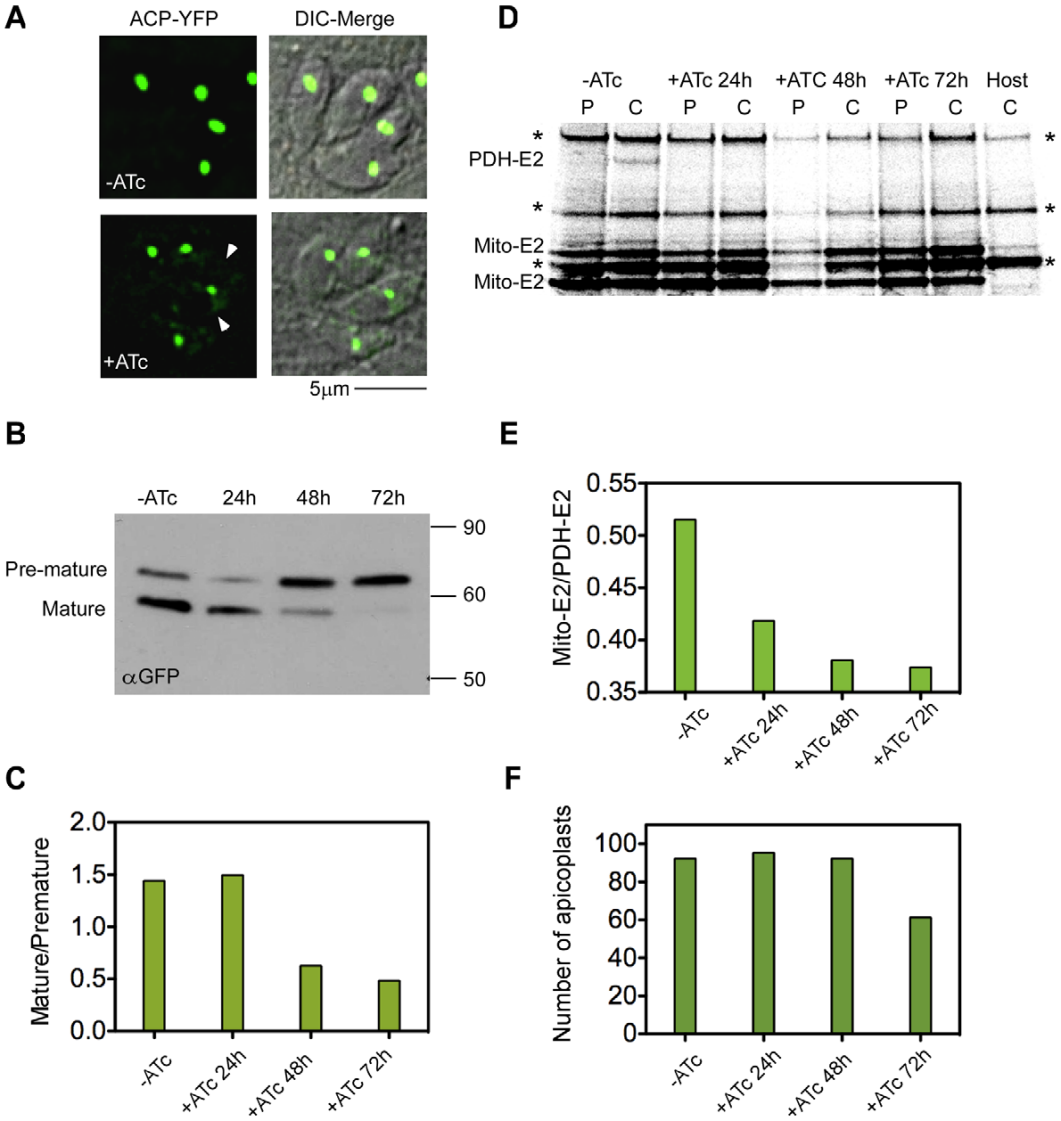
Loss of PPP1 results in apicoplast protein import defect. (A) To study apicoplast biogenesis in the *PPP1* mutant we engineered a reporter by tagging the ACP locus with YFP. No overt apicoplast division defects were observed upon 48 h ATc treatment. However, note accumulation of fluorescence outside of the apicoplast in treated parasites after 48 h (arrowheads). (B) Western blot analysis following the maturation of ACP-YFP in the (Tet)PPP1(HA) mutant. Parasites were grown in ATc for the times indicated. Anti-GFP antibody was used to detect ACP-YFP. Note the pronounced loss of the mature form of the protein. (C) Bands in B were quantified by densitometry and the ratio of mature to premature protein is shown for each time point. (D) Pulse-chase analysis of protein synthesis and post-translational lipoylation of apicoplast (single band labeled PDH-E2) and mitochondrial proteins (two bands marked mito-E2). Parasites were metabolically labeled as detailed in [13] and lipoylated proteins were isolated by immunoprecipitation using a specific antibody. One hour of labeling (Pulse, P) was followed by a two hour of chase (C) resulting in import and lipoylation of PDH-E2 in non treated parasites. Lipoylation is lost upon ATc treatment. Data shown are representative of four independent experiments. Bands labeled with an asterisk represent lipoylated host cell proteins (note that these bands are observed in host-only controls (Host, C). (E) Bands in D were quantified by densitometry and the ratio of lipoylated PDH-E2 over mito-E2 (upper mitochondrial band) is shown. (F) (Tet)PPP1(HA) parasite were grown in ATc as indicated and plastids were counted in 100 four-parasites vacuoles for each sample. Y-axis shows the percent of parasites that show a clearly identifiable apicoplast.

Having demonstrated that PPP1 is essential for growth, we were next interested to examine its specific biological role. Based on the putative PPC location we hypothesized that it may act in apicoplast biogenesis. To be able to follow organelle biogenesis, the luminal acyl carrier protein (ACP) was tagged with YFP directly in its locus in the mutant line, (Tet)PPP1(HA), to establish a native marker. Our western blot analysis suggested complete depletion of PPP1 between 24 h and 48 h of ATc treatment (Figure 7A). We therefore imaged ACP-YFP parasites at 48 h of growth in ATc. We observed intact plastids of typical shape in essentially all parasites (Figure 8A). This finding argues against a role of PPP1 in apicoplast division which produces aberrant plastid morphology and immediate loss of apicoplasts due to unequal segregation [55]. However, we noted that the intensity of the ACP-YFP signal in the organelle appeared to be reduced, and we observed an accumulation of the reporter protein outside of the plastid (Figure 8A arrowhead). This phenotype was observed previously in mutants of import components and suggested a protein import defect. We therefore next studied the maturation of ACP in this mutant in the presence and absence of ATc. Like many apicoplast proteins, ACP is synthesized with a bipartite N-terminal signal, which is cleaved upon reaching the organelle lumen, and this modification depends on import of the protein into the lumen of the organelle [13], [21], [32]. In cells grown in the absence of ATc, two bands are detected by western blot corresponding to the premature and mature protein forms (Figure 8B -ATc). In contrast, cells grown for 48 hours in ATc show a pronounced reduction of the mature form with concomitant accumulation of premature ACP-YFP (Figure 8B,C 48 h). We no longer detected the mature form after 72 hours of ATc treatment (Figure 8B,C 72 h). These observations suggested that loss of PPP1 results in an import defect.

As an independent measure of protein import, we determined the level of lipoylation of the apicoplast pyruvate dehydrogenase E2 subunit (PDH-E2). This was measured in real time by pulse-chase labeling after ATc treatment. Proteins are then immunoprecipitated with an antibody specific for lipoic acid. Protein import is a prerequisite for lipoylation of PDH-E2, as the process requires two apicoplast resident enzymes, and the precursor molecule octanoyl-ACP, which is synthesized de novo in the lumen of the organelle by the type II fatty acid synthesis system [3], [13], [32], [56]. We observed lipoylated PDH-E2 in lysate of untreated cells (Figure 8D, -ATc, C). After 24 hours under ATc treatment, the level of lipoylated PDH-E2 was dramatically reduced, and after 2 days was no longer detected (Figure 8C, +ATc, C and 8D 24 h/48 h). At the same time, lipoylation of mitochondrial proteins remained unchanged (Figure 8D mito-E2 bands).

It was previously documented that ablation of proteins involved in apicoplast import will ultimately result in organelle loss [13], [32]. Using the ACP-YFP marker, we scored the number of plastids at different time points of ATc treatment. At 24 hour and 48 hour time points, apicoplast numbers are comparable to the untreated control (over 92%). In contrast, at the 72 hour time point, we detect plastids in 61.25% of parasites (Figure 8E). This timing is similar to our previous observation for the *TgDer1* mutant, a periplastid component of the organellar protein import machinery [13]. Comparing the timing of our various measurements we find that loss of import precedes the loss of the organelle and parasite death. We note that after 72 h growth in the presence of ATc, organelles other than the plastid (such as the mitochondrion, micronemes and rhoptries) were indistinguishable from untreated or parental parasites (data not shown).

## Discussion

The apicoplast has exceptional potential as a target for anti-apicomplexan drugs. Its evolutionary history provides a large number of biochemical and cell biological mechanisms that are either absent from the human host or highly divergent. Many of these mechanisms are essential for parasite growth, either directly by providing an essential metabolite to the parasite cell, or indirectly by maintaining the organelle and its genome. To date, we have only scratched the surface by studying a small set of targets that were more readily identifiable due to the fact that they are well studied in chloroplasts. Many more targets are likely yet to be discovered. An important step on the way is to define the proteome of the apicoplast. Foth and coworkers took advantage of the presence of the bipartite signal found in many proteins destined to secondary plastids. They developed a prediction strategy for *in silico* identification of apicoplast proteins, which predicted 466 potential plastid proteins in *P. falciparum* [33]. More recently functional studies have shown that a significant proportion of apicoplast proteins lack such a signal, or feature a recessed signal peptide that prevents identification by this algorithm [9], [13], [24].

The genomes of several chromalveolates have been recently sequenced. This provides an important opportunity as many of these organisms harbor chloroplasts that share their ancestry with the apicoplast, and their targeting elements are more readily predicted [46], [57]-[59]. In a recent study, Moog and coworkers used these resources together with evidence for functional conservation, gene duplication and targeting signal prediction to search for PPC proteins in the diatom *P. tricornutum* [48]. Similarly, we chose to intersect data from different sources, weighing the cell cycle expression profiles and the molecular phylogenies of candidate proteins. Our search led to the identification of eleven apicoplast proteins (Table 1). Of those, five have homologs in *P. falciparum*, for which a bipartite targeting signal can be computationally predicted using PlasmoAP [33](Table 1), and one has a homolog in the recent study by Moog et al (PPP1). The remaining six are previously unpredicted apicoplast proteins, which reinforces the importance of the use of novel and varying strategies to enlarge the pool of known plastid components. Importantly, all bioinformatics avenues explored to date produced large numbers of both false positive and false negative assignments. Thus the importance of experimental validation cannot be overstressed at this point.

In this study we focused on genes which have mRNA abundance peaks in the G1 phase of the tachyzoite cell cycle [36]. Our observation that apicoplast proteins are enriched in this group (see Materials and Methods) supports the predictive value of this approach for Apicomplexa, and suggests expression profiling as a potential approach for similar studies on other organelles, or in related organisms. This is also supported by the non-apicoplast localizations we observed. For example, identification of five nuclear proteins adds to the number of genes that support the original report of a G1 wave of DNA replication genes [36]. Moreover mRNAs coding for proteins destined to the organelles involved in invasion (micronemes, rhoptries and dense granules) are known to peak in the S/M phase [36], and those were markedly absent from our dataset. A live imaging study by Nishi and coworkers also supports the notion of the ordered timing of organelle genesis in *T. gondii*, proposing a model of discontinuous synthesis and targeting of organellar proteins [60].

Following this idea further, we used the now enlarged pool of confirmed apicoplast proteins (Table S1) to ask whether their mRNA expression profiles correlates with apicoplast biological features. We noticed two expression clusters: for 23 genes the expression was highly focused towards the end of G1 while 36 genes showed a broader wave across this cell cycle phase (Table S1). Interestingly, the putative promoters (−2 kb to +0.1 kb) of the focused set show a common DNA sequence motif by FIRE analysis (Figure S6). This motif (GAGACA) is near identical to motif #8 (AGAGACA) reported by Behnke and coworkers to be enriched in the G1 phase [36]. Such a consensus motif does not emerge with statistical significance from the genes encoding non-apicoplast proteins, or when 28 genes are picked at random from either the list of 369 genes or the entire genome. Identifying a potential common regulator of expression for apicoplast proteins could provide additional avenues to identify apicoplast proteins.

One of our main goals was to identify truly novel apicoplast proteins, and the filtering pipeline was adjusted accordingly. As a result, 8 of the newly identified proteins are annotated as predicted hypothetical proteins, half of them are conserved among all apicomplexans. Now we are faced with the challenge of deducing their biological roles, and establishing those which may be suitable future drug targets. We attempted direct gene knock outs in the Δ*Ku80* background, using recombineered cosmids, and found that 4 of our new genes are likely indispensable for *T. gondii*, while 2 others are not essential for growth *in vitro* (Table 1). A robust and rapid system to generate conditional mutants was needed to pursue this further. We constructed a parasite strain that combines tight control of gene expression using the tetracycline inducible transactivator (TATi) system [51] with a low background of non-homologous recombination [37], [38]. We confirmed the efficiency of locus manipulation in this strain by epitope knock in, and localization of 30 proteins in this study. This strain can also be used to reliably construct mutants by promoter replacement. In this study we isolated two conditional mutants, and we note that using the same reagents we were able to generate six additional mutants in independent studies on apicoplast protein import and genome maintenance (Swati Agrawal, Sarah Reiff, LS, JD and BS unpublished). Promoter replacement not only allows for higher throughput, but also permits the study of genes that are difficult to express as ectopic copies (either due to large size, or due to the loss of biological activity upon epitope tagging).

Among the newly identified proteins, PPP1 stands out as a gene unique to the red algal lineage, and that in cryptomonads is encoded in the nucleomorph. Proteins encoded by the nucleomorph are destined to two main locations: the lumen of the plastid and the periplastid compartment [61]. Our light and electron microscopy demonstrated that PPP1 is a protein of the apicoplast periphery (Figure 3). Pinpointing a particular compartment of the periphery is difficult due to the minute size of the apicoplast. Efforts to use a split GFP assay [32] were hampered by our inability to construct suitably tagged versions of PPP1. Our results for the *T. pseudonana* homolog (Figure 3C), as well as the recent localization of a *P. tricurnitum* homolog [48] to the diatom plastid PPC, make residence in this compartment in apicomplexans very likely.

PPP1 lacks a typical apicoplast leader sequence at its N-terminus. However, it possesses a hydrophobic sequence at amino acids 86-106 that could serve as a recessed signal. Such recessed signals were previously described for apicoplast proteins such as CDC48_ Ap_ and Der1_Ap_ [13], both likely PPC residents. In diatoms, it is thought that a conserved phenylalanine in the leader of luminal proteins prevents cleavage prior to reaching the lumen. Proteins lacking this feature presumably lose their transit peptide in the PPC and thus remain in this compartment [57]. Such a residue is not obvious in apicomplexan plastid proteins, and further studies are required to dissect these targeting elements. In this context it is interesting to note that all *T. gondii* PPC proteins seem to manifest a patchy staining pattern (Figure 2B, 3A and [13]), rather than the more uniform staining shown for other peripheral proteins ([23], [24] and *001270* here). Polarized accumulation is particularly evident on electron-micrographs labeled for PPP1. Overall this is reminiscent of the blob-like structure of the diatom plastid, where PPC markers show a concentrated staining close to the center of the organelle, rather than an even circumferential distribution [46]. In a recent study, Tawk and coworkers showed that phosphatidylinositol 3-monophosphate (PI3P) is involved in the trafficking of ER derived vesicles to the apicoplast [27]. The use of a PI3-kinase inhibitor resulted in polarized accumulation of membranes in the plastid periphery [27]. These interesting observations together with the localization of PPP1 may point to a polarization of apicoplast protein import. How this particular structure serves the function of the organelle remains to be defined.

Based on proteomic evidence PPP1 is one of the most abundant apicoplast proteins. We show here that it plays an important role in apicoplast and parasite biology. Depletion of PPP1 results in the rapid demise and death of *T. gondii*. Our studies link PPP1 function to protein import. We demonstrate the loss of transport-dependent post-translational modifications of luminal apicoplast proteins in the absence of PPP1. The timing of the loss of import when compared to the loss of organelle (the ultimate phenotype of all apicoplast biogenesis mutants [3], [13], [32], [55]) suggests import as the primary defect of the PPP1 mutant. Based on the current model for apicoplast protein import [62], a role for PPP1 may be considered for three steps of the import process: it may interact with the recently described ERAD system to help cargo to enter the PPC, it could chaperone cargo proteins while they cross this compartment, or it might facilitate their interactions with the presumptive TOC complex in order to leave the PPC on the way to the lumen.

The second likely PPC protein that we identify here is ATrx2 a conserved thioredoxin-like protein. Interestingly, the ATrx2 CXXC sequence differs from the canonical motif. The classic reductive-type Trxs share a C(G/P)PC motif, and protein disulfide isomerases and the oxidative type DsbA protein have conserved a C(P/G)HC sequence (reviewed in [63]), whereas ATrx2 and its homologs contain a C(E/D)(H/Y)C sequence (Figure S5). Studies have shown that the central residues of the motif define the redox potential of Trx proteins, and control their ability to interact with substrate proteins and to isomerize disulfides [45]. What might be the role of Trx in this compartment, which is derived from the cytoplasm of the algal endosymbiont? Several lines of evidences connect the redox control with chloroplast protein import (reviewed in [64]). Specifically components of the TOC machinery appear to be subjected to regulation through disulfide bridge formation in domains exposed to the cytoplasm [65], [66], which, in the case of secondary plastids, is equivalent to the PPC. A family of non-canonical CXXC containing Trx proteins was recently reported in the chloroplast of *Arabidopsis thaliana*. At least two of them are distributed between the lumen and membranous fraction of the chloroplast [63]. It is therefore tempting to hypothesize that ATrx2 and PPP1 not only share their localization and peculiar phylogenetic distribution but that both may play a role in the same pathway of apicoplast protein import.

Our initial study was focused on developing tools suitable for a large-scale interrogation of apicoplast function. We studied a subset of 50 genes, which produced 11 new apicoplast proteins including several with essential function. The computational and experimental pipeline assembled here has shown power and throughput and should allow us to assemble a comprehensive and prioritized list of potential apicoplast intervention targets.

## Materials and Methods

### Parasite and diatom culture and genetic manipulation

Parasites were grown in hTERT-BJ1 (clontech) cells in supplemented Dulbecco's modified Eagle's medium [67]). Parasite cloning and plaque assays were performed in human foreskin fibroblasts (HFF). For the selection of stable transgenic lines, drugs were added as follow: 1 µM pyrimethamine added one day after transfection for one week, 20 µM chloramphenicol added the day of transfection for three weeks, 5 µM FUDR added two days after transfection for one week. To repress the regulated promoter, parasites were grown in the presence of 0.5 µM anhydrotetracycline (ATc).

*Thalassiosira pseudonana* (Hustedt) Hasle et Heimdal CCMP1335 was grown in an artificial seawater medium (EASW) according to the North East Pacific Culture Collection protocol (http://www3.botany.ubc.ca/cccm/NEPCC/esaw.html) at 18°C under constant light. Where indicated, NaNO_3_ was omitted from the medium (nitrogen-free medium) or replaced by 0.55 mM NH_4_Cl (ammonium medium).

### Identifying apicoplast protein candidates using bioinformatics resources

The mRNA abundance dataset generated by [36] was interrogated using GeneSpring GX 11..5 (Agilent). We searched for genes with periodic expression patterns that matched each of the 20 “baits” (Table S1), using Pearson correlation at 0.05 FDR. A group of 369 candidate genes (Table S2) was composed where each gene showed high correlation to at least one of the genes in the training group.

We next screened the first 200 genes in this list using a scoring system to filter candidates based on molecular phylogeny. We used tBLASTn to compare each of our candidate amino acid sequences against translated nucleotide databases. Only hits with E-value scores smaller than 1×10^−4^ were considered, and the following scores were given to candidates whose homologs were found in the following organisms (organellar genomes): Cyanobacteria (10); *Cryptosporidium* (−1); No *Cryptosporidium* (1); *Plasmodium* (1); distribution of homologs among eukaryotes outside Archaeplastida (−1 to -5, depending on number of organisms and level of similarity and coverage); none of the latter (1); distribution among Archaeplastida (1 to 5); nucleomorph genome (5).

### Multiple alignment and phylogenetic analyses

We generated multiple sequence alignments using ClustalX. The pairwise alignment parameters included a gap opening penalty of 35, and gap extension penalty of 0.75. Sequences that were used for alignments were identified on publicly available databases (see results section), and their accession numbers are provided in Table S5. 140 (PPP1) or 99 (ATrx2) unambiguously aligned amino acid positions were used for analysis using Jalview (full alignments available on request). For PPP1 the alignment presented in Figure 4 was used to generate a bootstrapped neighbor joining in ClustalX with 999 repetitions. The tree was visualized with TreeView.

### Sequence analyses and gene cloning

Prior to tagging, the computationally predicted gene models for each of the candidates were re-assessed manually, with particular attention paid to the predicted stop codon. We scrutinized expressed sequence tags (ESTs), peptides identified through proteome-analyses, and the presumptive position of active promoters as identified by chromatin modification (all accessed through ToxoDB). We determined alternative gene models for 4 of the 57 candidates. No *in silico* evidence was available at the time of assessment to dispute the predicted gene models for the remaining 54 genes. Data from deep sequencing mRNA became available at later stages of our study, and supported the predicted C-terminal assignment for 53 of our models (Table S3),

As the size and processing pattern of internally tagged PPP1 detected by western blots was inconsistent with the gene model, we experimentally determined its N-terminus. We tested three alternative translation start sites for which the respective coding sequences were amplified from cDNA, cloned into an expression vector that fuses a C-terminal myc epitope tag [32] and transfected into parasites. Immunofluorescence showed that only the start codon at position 1,435,362 on chromosome TGME49_chrV resulted in apicoplast targeting (data not shown). Western blot analysis of this strain resulted in bands of sizes indistinguishable to those detected from the endogenously HA-tagged protein (not shown). Similarly, for *039680* we experimentally established an alternative start site from the predicted gene model at position 831330 on chromosome TGME49_chrVI. The new gene models are reported in user comments on the corresponding gene pages in ToxoDB and were submitted to genbank (JN053049, JN053050). In both cases, the coding sequence was amplified by PCR from *T. gondii RH* cDNA using primers introducing flanking BglII or BclII and AvrII or XbaI restriction sites, and products were cloned into BglII and AvrII sites in *pDT7S4myc* [32].

To modify loci with a triple HA epitope tag, the vector *p3HA.LIC.DHFR* (generously shared by Huynh and Carruthers [38]) was modified to replace the DHFR (Dihydrofolate reductase) cassette (HindIII and NotI) with a chloramphenicol acetyl-transferase (CAT) expression cassette excised with the same enzymes from *pTUB5CAT* [68]. Next, the PacI site found within the CAT-ORF was mutated by site directed mutagenesis (Stratagene) to render the PacI site found within the LIC (ligation independent cloning) sequence unique, generating *p3HA.LIC.CATΔpac*. Targeting sequences were amplified by PCR from *T. gondii* RH genomic DNA and products were LIC-cloned into *p3HA.LIC.CATΔpac* as described previously [38]. Positive clones were isolated using primer 1595 in combination with the specific forward primer by PCR screen and confirmed by sequencing. *pLIC ACP YFP* was generated similarly in *pLIC YFP DHFR* [38].

For complementation and UPRT targeting, PPP1 was first sub-cloned from *pDT7S4PPP1.1myc* into *pBTTgTPTty* [6] using BglII and AvrII sites. Then the entire *TUB_PPP1_Ty* cassette was excised with BamHI and SpeI and cloned into the BglII and AvrII sites in *pUPRT-KO* (a gift of Brooke and Gubbels). The resulting vector, *pUPRT_(TUB)PPP1Ty* was transfected into (Tet)PPP1(HA) and clones were isolated after FUDR (5 µM) selection, and tested for expression of PPP1Ty by IFA.

For promoter replacements *pDT7S4myc* [32] was modified such that the 3' targeting flank (the coding sequence beginning with the initiation codon) was cloned between BglII and AvrII, and the 5' flank (upstream of predicted promoter region) was cloned between two NdeI sites found in the 5'UTR of the DHFR selection cassette.

Promoter regions were predicted based on the ChIP on chip data [69] viewed through ToxoDB. Accordingly, the 1.7 kb fragment found in positions 1,434,003 to 1,435,362 on TGME49_chrV was amplified with NdeI sites for PPP1, and the 0.7 Kb upstream of position 831330 on chromosome TGME49_chrVI was amplified with MseI sites for *039680*. The resulting vectors were linearized with AvrII (PPP1) and BstBI (*039680*).

All primers used are found in Tables S3 and S4.

### Microscopy

Immunofluorescence assays were performed as previously described [70]. We used anti-HA antibodies (Roche) at a dilution of 1∶200, anti-CPN60 [13] at 1∶1000, anti-ATrx1 [23] at 1∶1000, anti-Ty BB2 hybridoma supernatant [71] at 1∶5, anti–Myc (Pierce) at 1∶100, anti-GFP (Roche) at 1∶200, anti-TgMys [72] at 1∶1000, antiROM4 [70] at 1∶100, anti-IMC3 [73] at 1∶500. Fluorescence images were acquired using a Delta Vision microscope as described [13].

For cryo-electron microscopy, infected cells were fixed in 4% paraformaldehyde/0.05% glutaraldehyde (Polysciences Inc.) in 100 mM PIPES buffer. Samples were then embedded in 10% gelatin and infiltrated overnight with 2.3 M sucrose/20% polyvinyl pyrrolidone in PIPES at 4°C. Samples were frozen in liquid nitrogen and sectioned with a cryo-ultramicrotome. Sections were probed with anti-HA antibody followed by a rat secondary antibody conjugated to 18 nm colloidal gold, stained with uranyl acetate/methylcellulose, and analyzed by transmission EM as described previously [74].

Confocal fluorescence microscopy of *T. pseudonana* was performed using an inverted Zeiss LSM 510 laser scanning microscope (Jena, Germany). Fluorescent signals were detected for GFP (Argon laser, 488 nm) using a 505/550-nm bandpass filter and chloroplast auto-fluorescence (HeNe laser, 543 nm) using a 585 nm long pass filter in the multitrack mode of the microscope. Cells were immobilized for microscopy by a thin slice of 1% Agarose dissolved in EASW medium.

### Western blotting and pulse-chase analyses

Western blotting was performed as previously described [32]. We used anti-HA antibodies (Roche) at a dilution of 1∶100, anti-Tubulin [75] at 1∶1000, anti-GFP (Roche) 1∶200 , anti-Myc (Pierce) 1∶100.

Pulse-chase analyses were performed as described previously [13], [32] with the modification that experiments were performed in T25 flasks.

## Supporting Information

Figure S1 Fluorescence microscopy analysis of parasites expressing 11 endogenously HA-tagged (green) luminal (A) or peripheral (B) plastid proteins, co-stained with the luminal marker CPN60 (red). Merge of both antibodies, DIC and DAPI staining are shown. Numbers reflect the ToxoDB gene ID as detailed in the Results section. Scale bar is 5 µm.
(TIF)

Figure S2 Fluorescence microscopy analysis of parasites expressing 19 endogenously HA-tagged (green) proteins, co-stained with markers for various compartments (red): surface (antiROM4); apicoplast (CPN60); Golgi (GRASP-RFP); Nucleus (DAPI); IMC (IMC3).
(TIF)

Figure S3 (A) Schematic representations of homologous recombination events in the Ku80KO-line genome driven by *mTOXOW30* cosmid to disrupt *PPP1* gene. Primers used for PCR are also indicated with the corresponding lanes numbers for panel B. (B) PCR analysis of 7 representative stable clones established after chloramphenicol selection, showing no disruption of PPP1 ORF.
(TIF)

Figure S4 Schematic representation of the expected southern blot band-sizes based on the manipulation of the *PPP1* locus. Top (P, parental) shows the native locus as it is expected to be in the TATi*ΔTgKu80* line and the position of the probe used for southern. Middle (T, tagged) shows the modification upon tagging and corresponding new band-sizes. Bottom (PR, promoter replacement) shows the modification resulting in double modified locus and the corresponding new band-sizes.
(TIF)

Figure S5 (A) Schematic representation of the likely phylogenetic relationship among the members of chromalveolates (redrawn from [62] based on a phylogenetic analysis by 76. Keeling PJ, Burger G, Durnford DG, Lang BF, Lee RW, et al. (2005) The tree of eukaryotes. Trends Ecol Evol 20: 670–676. Names of phyla are shown in bold, those carrying plastids are further shown in italic font. Species used in the alignments of PPP1 and/or ATrx2 are listed below their respective phylum. (B) Multiple protein sequence alignment of the predicted Trx domain of the putative orthologues of ATrx2. Blue color gradient corresponds to percentage identity where deep blue is 100%. Size of black bars corresponds to level of consensus conservation.
(TIF)

Figure S6 (A) Graphs showing mRNA abundance profiles for the two expression waves identified for apicoplast encoding genes. (B) A common motif found by FIRE analysis in the putative promoter region of all the genes of the tight G1 wave.
(TIF)

Table S1 Total of confirmed apicoplast protein encoding genes with their use in this study and their mRNA periodic cluster data.
(PDF)

Table S2 369 G1 apicoplast cluster list of genes.
(PDF)

Table S3 57 genes chosen to be experimentally addressed, including primers, PCR products sizes and linearization sites.
(PDF)

Table S4 Other primers used in this study.
(PDF)

Table S5 Accession numbers for genes used in alignments or phylogeny.
(PDF)
