# Supplementary figures and images for: A Systematic Screen to Discover and Analyze Apicoplast Proteins Identifies a Conserved and Essential Protein Import Factor
Source: PLoS Pathog. 2011 Dec 1;7(12):e1002392. doi: 10.1371/journal.ppat.1002392 (PMC3228799; doi:10.1371/journal.ppat.1002392)

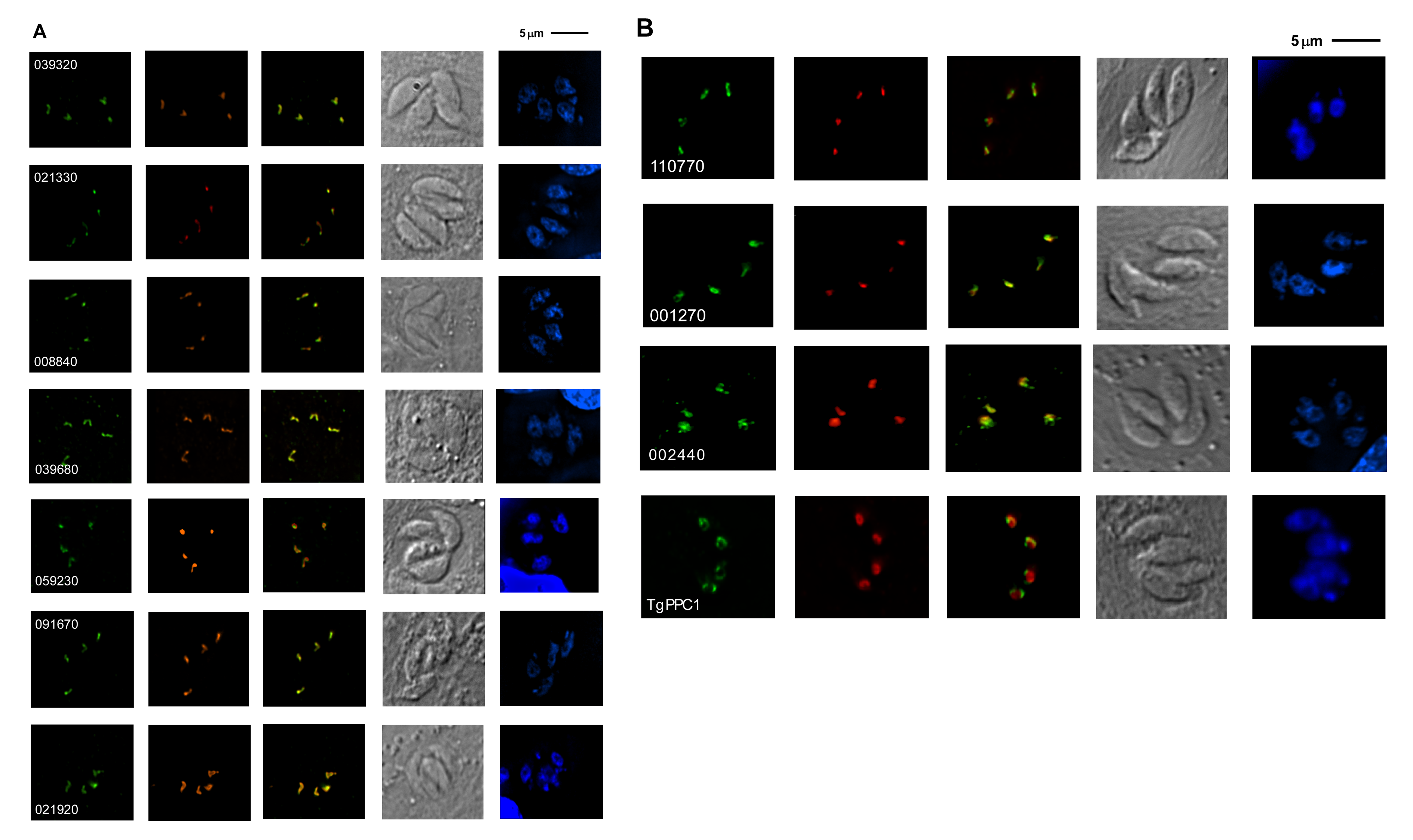

Supplement: Figure S1 — Fluorescence microscopy analysis of parasites expressing 11 endogenously HA-tagged (green) luminal (A) or peripheral (B) plastid proteins, co-stained with the luminal marker CPN60 (red). Merge of both antibodies, DIC and DAPI staining are shown. Numbers reflect the ToxoDB gene ID as detailed in the Results section. Scale bar is 5 µm. (TIF) [file ppat.1002392.s001.tif]

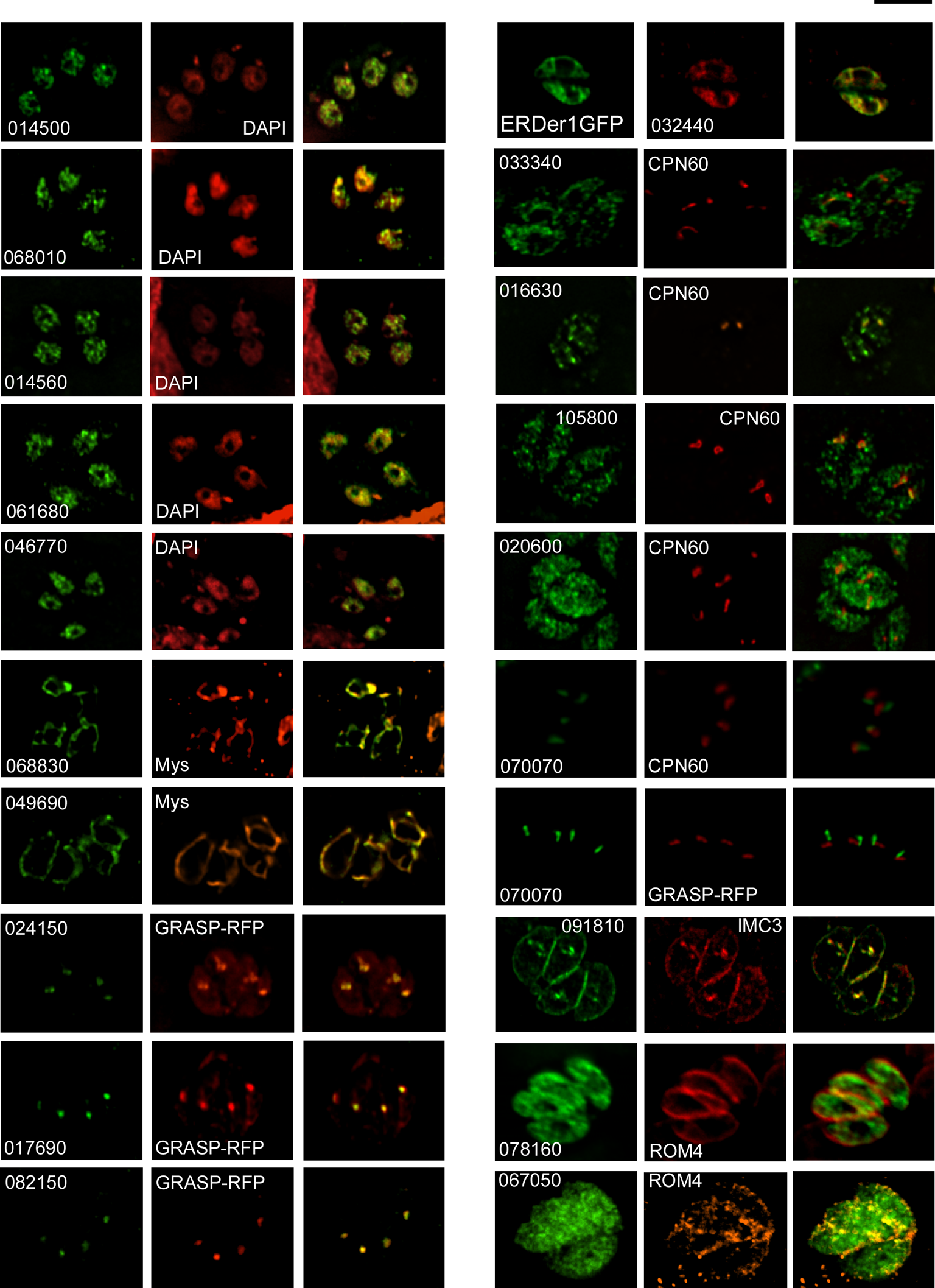

Supplement: Figure S2 — Fluorescence microscopy analysis of parasites expressing 19 endogenously HA-tagged (green) proteins, co-stained with markers for various compartments (red): surface (antiROM4); apicoplast (CPN60); Golgi (GRASP-RFP); Nucleus (DAPI); IMC (IMC3). (TIF) [file ppat.1002392.s002.tif]

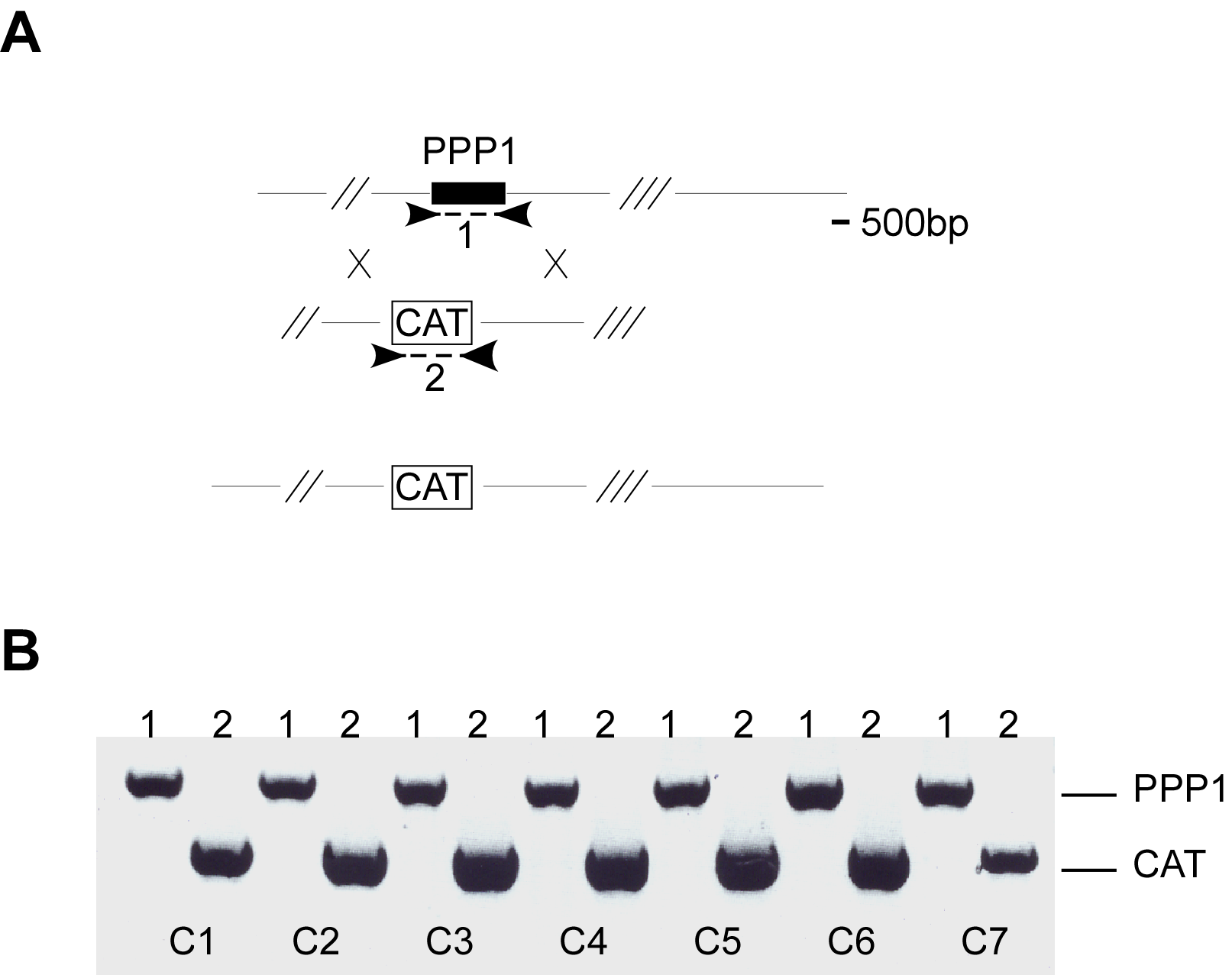

Supplement: Figure S3 — (A) Schematic representations of homologous recombination events in the Ku80KO-line genome driven by mTOXOW30 cosmid to disrupt PPP1 gene. Primers used for PCR are also indicated with the corresponding lanes numbers for panel B. (B) PCR analysis of 7 representative stable clones established after chloramphenicol selection, showing no disruption of PPP1 ORF. (TIF) [file ppat.1002392.s003.tif]

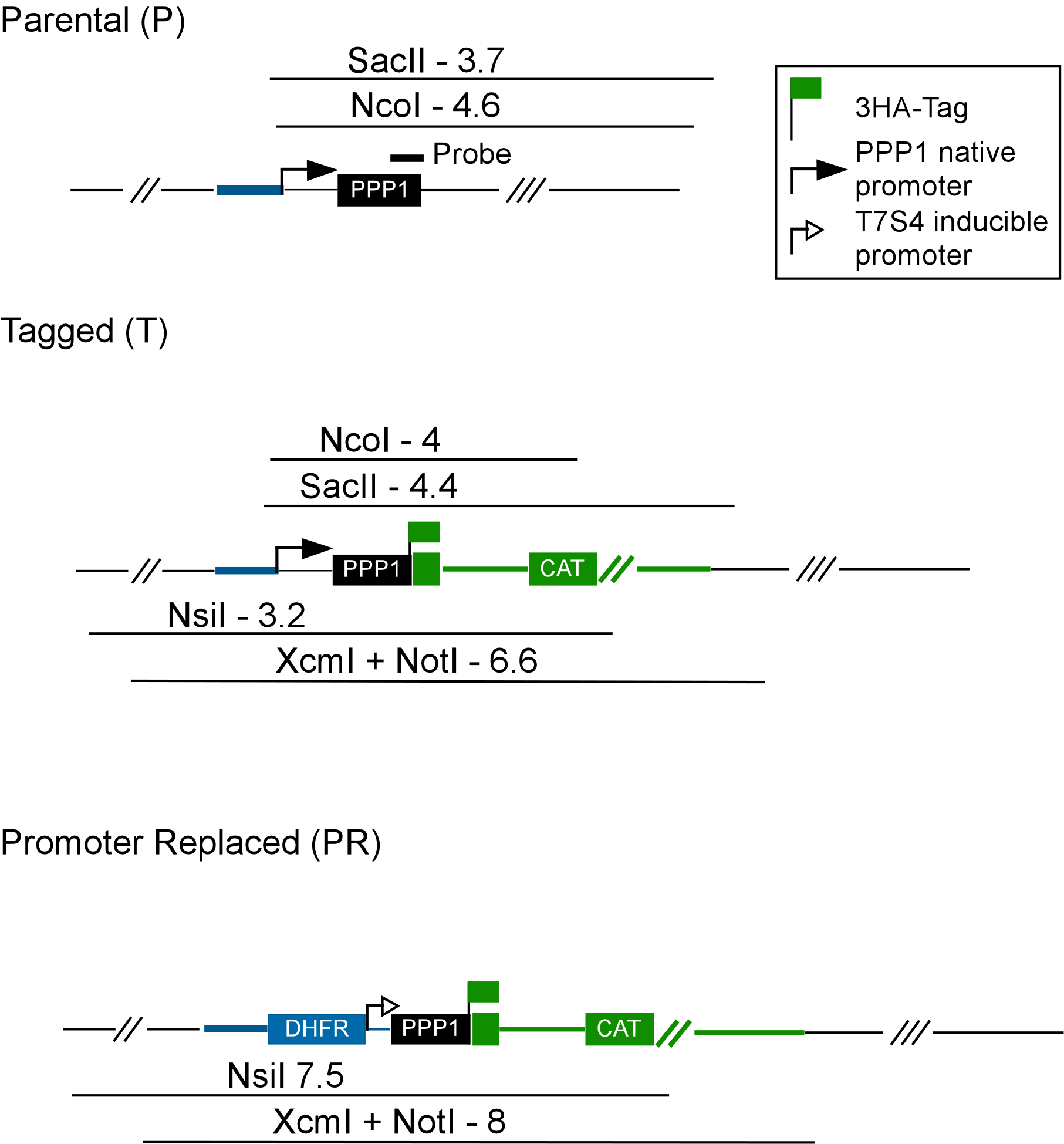

Supplement: Figure S4 — Schematic representation of the expected southern blot band-sizes based on the manipulation of the PPP1 locus. Top (P, parental) shows the native locus as it is expected to be in the TATiΔTgKu80 line and the position of the probe used for southern. Middle (T, tagged) shows the modification upon tagging and corresponding new band-sizes. Bottom (PR, promoter replacement) shows the modification resulting in double modified locus and the corresponding new band-sizes. (TIF) [file ppat.1002392.s004.tif]

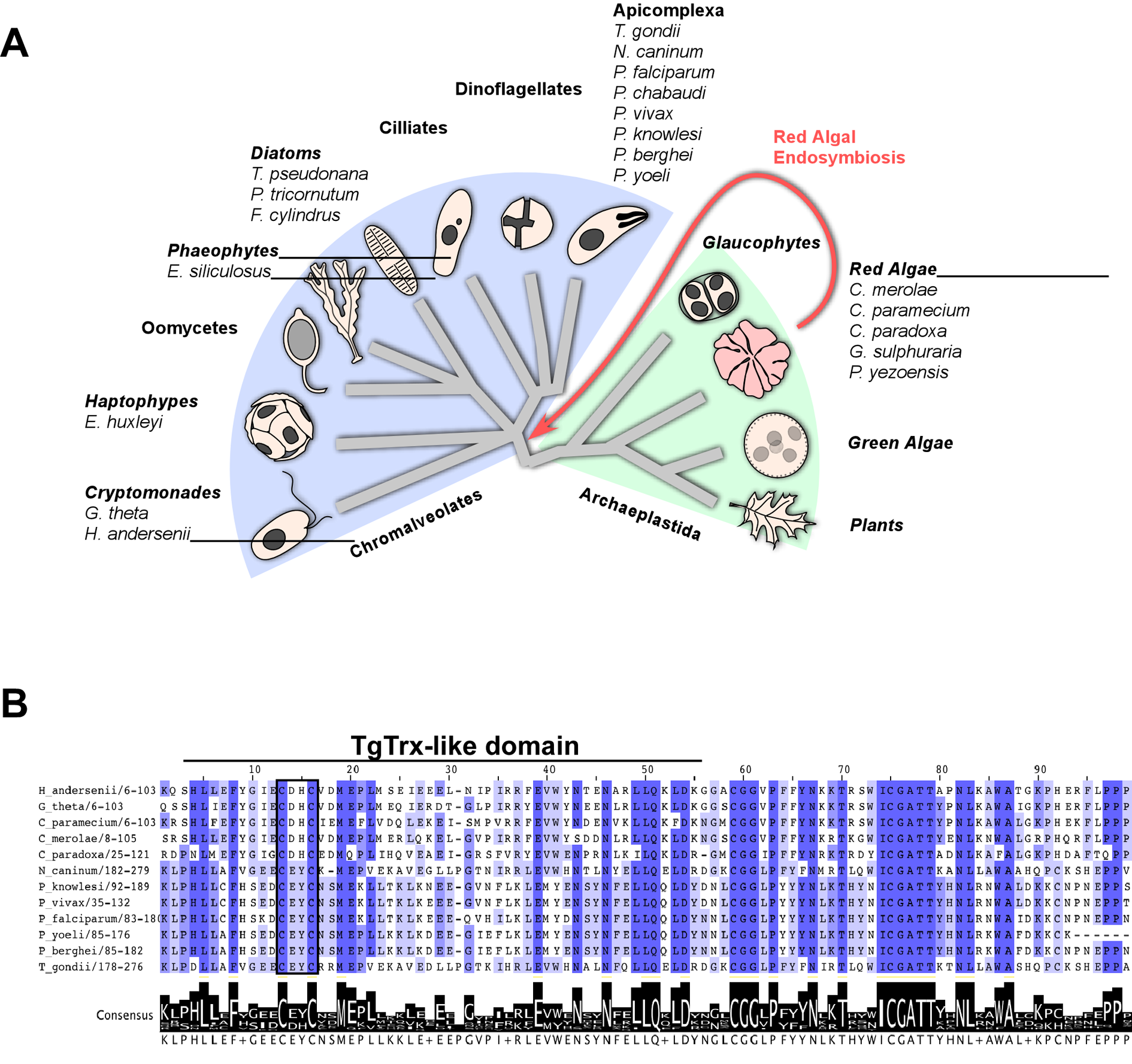

Supplement: Figure S5 — (A) Schematic representation of the likely phylogenetic relationship among the members of chromalveolates (redrawn from [62] based on a phylogenetic analysis by 76. Keeling PJ, Burger G, Durnford DG, Lang BF, Lee RW, et al. (2005) The tree of eukaryotes. Trends Ecol Evol 20: 670–676. Names of phyla are shown in bold, those carrying plastids are further shown in italic font. Species used in the alignments of PPP1 and/or ATrx2 are listed below their respective phylum. (B) Multiple protein sequence alignment of the predicted Trx domain of the putative orthologues of ATrx2. Blue color gradient corresponds to percentage identity where deep blue is 100%. Size of black bars corresponds to level of consensus conservation. (TIF) [file ppat.1002392.s005.tif]

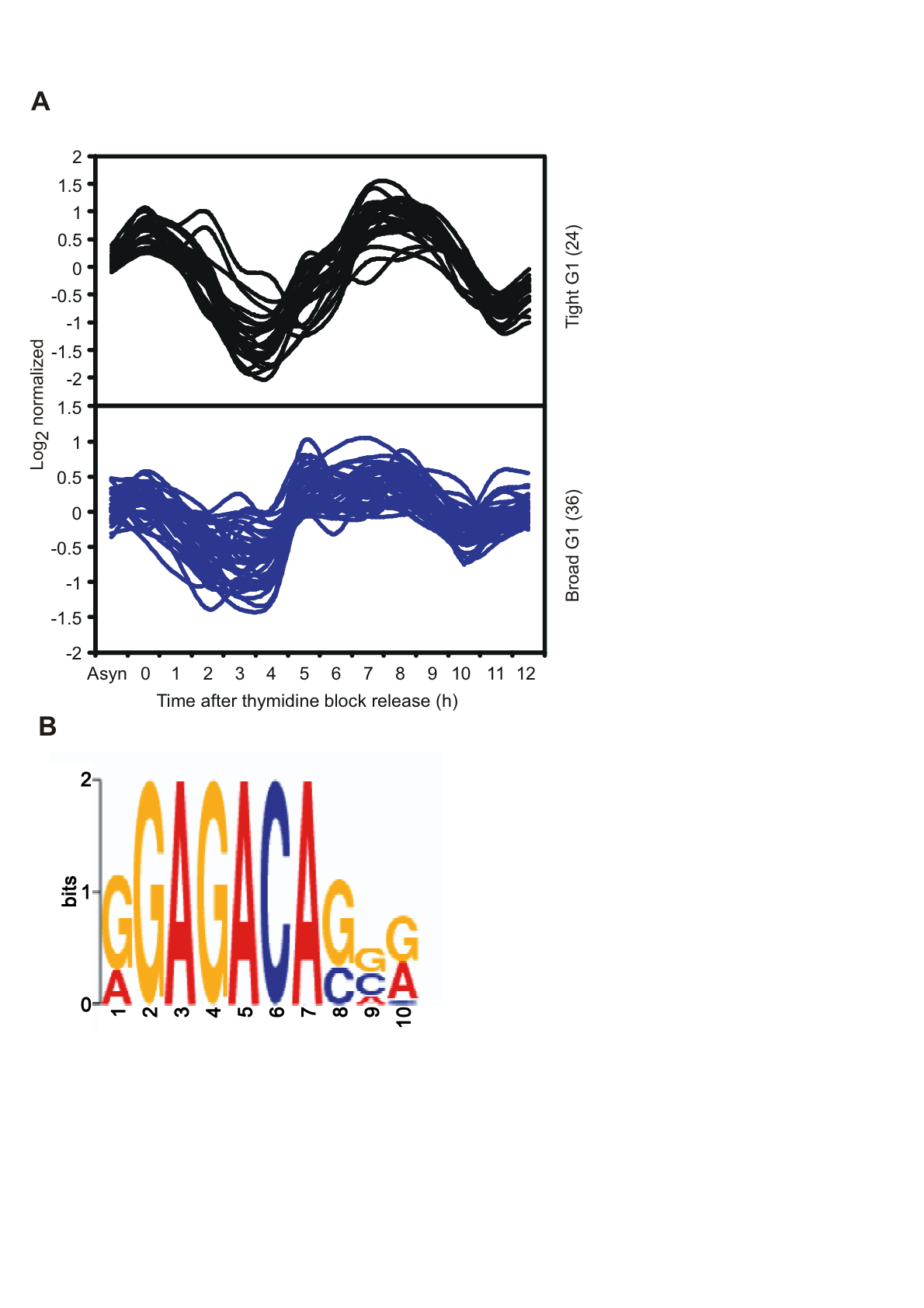

Supplement: Figure S6 — (A) Graphs showing mRNA abundance profiles for the two expression waves identified for apicoplast encoding genes. (B) A common motif found by FIRE analysis in the putative promoter region of all the genes of the tight G1 wave. (TIF) [file ppat.1002392.s006.tif]
