# Supplementary material for: A Systematic Screen to Discover and Analyze Apicoplast Proteins Identifies a Conserved and Essential Protein Import Factor
Source: PLoS Pathog. 2011 Dec 1;7(12):e1002392. doi: 10.1371/journal.ppat.1002392 (PMC3228799; doi:10.1371/journal.ppat.1002392)
Supplement: Table S1 — Total of confirmed apicoplast protein encoding genes with their use in this study and their mRNA periodic cluster data. (PDF) [file ppat.1002392.s007.pdf]

List of confirmed apicoplast proteins

Gene ID	Product Description	Apicplast process or pathway	Source	New name	Cluster
**20 genes used to seed cell cycle data to create the 369 G1 apicoplast list**					
TGME49_021330	DNA gyrase subunit A, putative	Genome maintanance	Confirmation - this study	TgGyrA	Broad G1
TGME49_064080	acyl carrier protein	Fatty acid sythesis	ACP, PMID: 9770490, PMID: 16920791	ACP	Tight G1 (15 genes) + pomoter element
TGME49_093590	3-oxoacyl-[acyl-carrier-protein] synthase, putative	Fatty acid sythesis	PMID: 12549938, PMID: 16467310	FabB/F	Tight G1 (15 genes) + pomoter element
TGME49_105980	dihydrolipoyl dehydrogenase protein, putative	Pyruvat metabolism	PMID: 17449654	PDH E3	Tight G1 (15 genes) + pomoter element
TGME49_059260	cell division protein, putative		PMID: 19450729, PMID: 17822404	FtsH	Broad G1
TGME49_025990	malonyl CoA-acyl carrier protein transacylase, putative	Fatty acid sythesis	PMID: 12549938	FabD	Tight G1 (15 genes) + pomoter element
TGME49_022020	phosphoglycerate kinase, putative		PMID: 17449654	Phosphoglycerate kinase II	Tight G1 (15 genes) + pomoter element
TGME49_006610	biotin requiring domain-containing protein / 2-oxo acid dehydrogenases acyltransferase catalytic domain-containing protein	Pyruvat metabolism	PMID: 17449654, PMID: 16778769	PDH E2	Tight G1 (15 genes) + pomoter element
TGME49_031890	3-oxoacyl-(acyl-carrier-protein) synthase III family protein, putative	Fatty acid sythesis	(PfKASIII), PMID: 12270624	FabH	Tight G1 (15 genes) + pomoter element
TGME49_072290	pyruvate dehydrogenase E1 beta subunit, putative	Pyruvat metabolism	PMID: 17449654, PMID: 15612915	PDH E1 beta	Tight G1 (15 genes) + pomoter element
TGME49_033500	triosephosphate isomerase, putative		PMID 17449654	Triosephosphate isomerase II	non periodic
TGME49_051930	enoyl-acyl carrier reductase	Fatty acid sythesis	PMID: 11239932, PMID: 17327670	FabI	Tight G1 (15 genes) + pomoter element
TGME49_099070	pyruvate kinase, putative	Pyruvat metabolism	PfPyKII, PMID: 19015045	Pyruvate kinase II	Broad G1
TGME49_009710	50S ribosomal protein L28, putative	Translation	PMID: 9770490	rpl28	Broad G1
TGME49_040600	TCP-1/cpn60 chaperonin family protein, putative	Iron-Sulfur cluster	PfCPN60, PMID: 14747157	CPN60	Tight G1 (15 genes) + pomoter element
TGME49_021320	acetyl-CoA carboxylase, putative	Fatty acid sythesis	PMID: 10557330	ACC1	Tight G1 (15 genes) + pomoter element
TGME49_112110	nucleoredoxin, putative		PMID: 18586952	Atrx	Broad G1
TGME49_045670	pyruvate dehydrogenase, putative	Pyruvat metabolism	PMID: 17449654, PMID: 15612915	PDH E1 alpha	Tight G1 (15 genes) + pomoter element
TGME49_066760	isocitrate dehydrogenase, putative		PMID: 17784785	ICDH2	Tight G1 (15 genes) + pomoter element
TGME49_061070	hypothetical protein	Metabolite import	PMID: 17449654, PMID: 17822404, PMID: 20036630	APT	Tight G1 (15 genes) + pomoter element
**Confirmed apicoplast genes included in the first 200 hits of the 369 G1 apicoplast list**					
TGME49_085700	hypothetical protein	Apicoplast proteins import	PMID: 19808683	Udf ap	Tight G1 (15 genes) + pomoter element

List of confirmed apicoplast proteins

TGME49_055680	YbaK / prolyl-tRNA synthetases associated domain containing protein	Isoprenoid synthesis	Nair et al; in press	YgbB	Broad G1
TGME49_053730	importin-alpha re-exporter, putative	Isoprenoid synthesis	Nair et al; in press	4-diphosphocytidyl-2-methyl-D-erithritol synthase	Broad G1
TGME49_089940	uroporphyrinogen decarboxylase, putative	Heme biosynthesis	PfHemE, PMID: 19523497	HemE (UROD)	Broad G1
TGME49_055690	2C-methyl-D-erythritol 2,4-cyclodiphosphate synthase domain-containing protein	Isoprenoid synthesis	Nair et al; in press	2C-methyl-D-erythritol 2,4-cyclodiphosphate synthase domain-containing protein (Sethu)	Broad G1
TGME49_008820	1-deoxy-D-xylulose 5-phosphate synthase, putative	Isoprenoid synthesis	Nair et al; in press	DXP synthase	Broad G1
TGME49_027970	DNA-binding protein HU, putative	Genome maintanance	TgHU, Rieff unpublished, PfHU, PMID: 18663012	TgHu	Tight G1 (15 genes) + pomoter element
TGME49_115640	biotin/lipoate A/B protein ligase domain-containing protein	Lipoic acid de novo sythesis	PfLipB, PMID: 18069893, PMID: 15225307	LipB	Broad G1
**Identified in this study**					
TGME49_091670	RNA helicase, putative	DNA-Replication and repair ?	This study		Broad G1
TGME49_059230	hypothetical protein	DNA-Replication and repair ?	This study		Tight G1 (15 genes) + pomoter element
TGME49_039320	hypothetical protein, conserved	Transcription ?	This study		Tight G1 (15 genes) + pomoter element
TGME49_110770	hypothetical protein		This study	TgAtrx2	Broad G1
TGME49_039680	hypothetical protein, conserved		This study		Broad G1
TGME49_002440	hypothetical protein		This study		
TGME49_001270	hypothetical protein		This study		Broad G1
TGME49_021920	hypothetical protein	Iron-Sulfur cluster ?	This study	NFU	Broad G1
TGME49_008840	ATP-dependent DNA helicase, putative	DNA-Replication and repair ?	This study	DNA dependaent DNA helicase	Broad G1
TGME49_087270	hypothetical protein	Apicoplast proteins import	this study, PtPPC1, PMID: 21498883	PPC1	Broad G1
**Confirmed apicoplast proteins not present in the 369 G1 apicoplast list**					
TGME49_055370	hypothetical protein	Apicoplast proteins import	PMID: 18757752	TIC20	Tight G1 (15 genes) + pomoter element
TGME49_086050	hypothetical protein	Apicoplast proteins import	PfTIC22, PMID: 19502580	TIC22	
TGME49_072390	hypothetical protein	Apicoplast proteins import	PtOmp85, PMID: 20042599	TOC75 (OMP85)	Broad G1
TGME49_081940	hypothetical protein, conserved	Apicoplast proteins import	PMID: 19808683	$Der1_{Ap}$	Tight G1 (15 genes) + pomoter element
TGME49_121640	cell division protein 48, putative	Apicoplast proteins import	PMID: 19808683	$Cdc48_{Ap}$	Broad G1
TGME49_114890	ubiquitin-activating enzyme E1, putative	Apicoplast proteins import	PfUbcE1, PMID: 19502583	Ubiquitin-conjugating enzyme E1	Broad G1
TGME49_095990	ubiquitin-conjugating enzyme E2, putative	Apicoplast proteins import	PfUbcE2, PMID: 19502583	Ubiquitin-conjugating enzyme E2	Broad G1

List of confirmed apicoplast proteins

TGME49_091980	ubiquitin-transferase domain-containing protein	Apicoplast proteins import	Agrawal unpublished	Ubiquitin-activating enzyme E1	Tight G1 (15 genes) + pomoter element
TGME49_003420	phosphopantethiene--protein transferase domain containing protein	Fatty acid sythesis	CpACPS-PPT, PMID: 16002647	ACPS-PPT	Broad G1
TGME49_121570	(3R)-hydroxymyristoyl ACP dehydrase, putative	Fatty acid sythesis	PMID: 18274777	FabZ	Tight G1 (15 genes) + pomoter element
TGME49_017740	oxoacyl-ACP reductase, putative	Fatty acid sythesis	PfFabG, PMID: 12646215	FabG	Tight G1 (15 genes) + pomoter element
TGME49_030990	hypothetical protein	Iron-Sulfur cluster	PMID: 18173393	Dephospho-CoA kinase	Broad G1
TGME49_015070	ferredoxin	Iron-Sulfur cluster	PMID: 11056177	Plant-type ferredoxin	Broad G1
TGME49_098990	ferredoxin NADP+ oxidoreductase, putative	Iron-Sulfur cluster	PMID: 11056177	Plant-type ferredoxin-NADPþ-reductase	Broad G1
TGME49_073960	10 kDa chaperonin, putative	Iron-Sulfur cluster	PfCPN20, PMID: 14747157	Chaperonin 20	Broad G1
TGME49_053900	delta-aminolevulinic acid dehydratase, putative	Heme biosynthesis	PfALAD, PMID: 14638682	HemB (ALAD)	Broad G1
TGME49_071420	porphobilinogen deaminase, putative	Heme biosynthesis	PfPBGD, PMID: 17962188	HemC (PBGD)	Broad G1
TGME49_058650	ferrochelatase, putative	Heme biosynthesis	PMID: 15307818	FC	Broad G1
TGME49_026400	lipoic acid synthase	Lipoic acid de novo sythesis	PMID: 12860390	Lipoate synthase LipA	Broad G1
TGME49_094900	DnaK family domain containing protein	Lipoic acid de novo sythesis	PMID: 19434237	Lipoic acid protein ligase A2 LiplA2	Broad G1
TGME49_097780	DNA gyrase subunit B, putative	Genome maintanance	PfGyrB, PMID: 17499371	GyraseB	Broad G1
TGME49_061800,TGME49_061920	helicase, putative/DNA polymerase I, putative	DNA-Replication and repair	PMID: 19402939	TgPrex	Broad G1
TGME49_018850	ribosomal protein S9, putative	Translation	PMID: 9770490	riboomal P S9	Tight G1 (15 genes) + pomoter element
TGME49_069190	glyceraldehyde-3-phosphate dehydrogenase		PMID: 17784785		Broad G1
TGME49_113120	RNA polymerase Rpb3/Rpb11 dimerisation domain-containing protein			RNA polymerase Rpb3/Rpb11 dimerisation domain-containing protein	Broad G1
