# Supplementary material for: A Systematic Screen to Discover and Analyze Apicoplast Proteins Identifies a Conserved and Essential Protein Import Factor
Source: PLoS Pathog. 2011 Dec 1;7(12):e1002392. doi: 10.1371/journal.ppat.1002392 (PMC3228799; doi:10.1371/journal.ppat.1002392)
Supplement: Table S2 — 369 G1 apicoplast cluster list of genes. (PDF) [file ppat.1002392.s008.pdf]

369 hits from expression profile based search

Gene ID	Product name	Status after phyletic filter
TGME49_064080	acyl carrier protein	included in other studies - ACP, PMID: 9770490, PMID: 16920791
TGME49_021350	hypothetical protein	included in other studies - Agrawal unpublished
TGME49_097240	hypothetical protein, conserved	included in other studies - Agrawal unpublished
TGME49_048140	hypothetical protein	included in other studies - Agrawal unpublished
TGME49_025350	hypothetical protein, conserved	included in other studies - Agrawal unpublished
TGME49_063240	hypothetical protein, conserved	included in other studies - Agrawal unpublished
TGME49_070220	hypothetical protein	included in other studies - Agrawal unpublished
TGME49_002290	hypothetical protein, conserved	included in other studies - Agrawal unpublished
TGME49_061070	hypothetical protein	included in other studies - APT, PMID: 17449654, PMID: 17822404, PMID: 20036630
TGME49_112110	nucleoredoxin, putative	included in other studies - Atrx1, PMID: 18586952
TGME49_040600	TCP-1/cpn60 chaperonin family protein, putative	included in other studies - CPN60, PMID: 14747157
TGME49_049180	bifunctional dihydrofolate reductase / thymidylate synthase	included in other studies - DHFR-TS, PMID: 12192007, PMID: 11257045
TGME49_093590	3-oxoacyl-[acyl-carrier-protein] synthase, putative	included in other studies - FabB/F, PMID: 12549938, PMID: 16467310
TGME49_025990	malonyl CoA-acyl carrier protein transacylase, putative	included in other studies - FabD, PMID: 12549938
TGME49_031890	3-oxoacyl-(acyl-carrier-protein) synthase III family protein, putative	included in other studies - FabH (PfKASIII), PMID: 12270624
TGME49_051930	enoyl-acyl carrier reductase	included in other studies - FabI, PMID: 11239932, PMID: 17327670
TGME49_009710	50S ribosomal protein L28, putative	included in other studies - L28, PMID: 9770490
TGME49_053730	importin-alpha re-exporter, putative	included in other studies - Nair et al; in press
TGME49_055690	2C-methyl-D-erythritol 2,4-cyclodiphosphate synthase domain-containing protein	included in other studies - Nair et al; in press
TGME49_008820	1-deoxy-D-xylulose 5-phosphate synthase, putative	included in other studies - Nair et al; in press
TGME49_045670	pyruvate dehydrogenase, putative	included in other studies - PDH E1, PMID: 17449654, PMID: 15612915
TGME49_006610	biotin requiring domain-containing protein / 2-oxo acid dehydrogenases acyltransferase catalytic domain-containing protein	included in other studies - PDH E2, PMID: 17449654, PMID: 16778769
TGME49_121670	mannosyltransferase, putative	included in other studies - PfGPI mannosyltransferase-II, PMID: 17977514
TGME49_089940	uroporphyrinogen decarboxylase, putative	included in other studies - PfHemE, PMID: 19523497
TGME49_115640	biotin/lipoate A/B protein ligase domain-containing protein	included in other studies - PfLipB, PMID: 18069893, PMID: 15225307
TGME49_095990	ubiquitin-conjugating enzyme E2, putative	included in other studies - PfUbcE2, PMID: 19502583
TGME49_022020	phosphoglycerate kinase, putative	included in other studies - PGKII, PMID: 17449654
TGME49_033500	triosephosphate isomerase, putative	included in other studies - PMID 17449654
TGME49_018840	mismatch repair protein, putative	included in other studies - PMID: 19291232
TGME49_085700	hypothetical protein	included in other studies - PMID: 19808683
TGME49_053290	valyl-tRNA synthetase, putative	included in other studies - PMID: 20374492
TGME49_066730	leucyl-tRNA synthetase, putative	included in other studies - PMID: 20374492
TGME49_020350	lysyl-tRNA synthetase, putative	included in other studies - PMID: 20374492
TGME49_088700	chromosome segregation protein smc1, putative	included in other studies - SMC1, PMID: 16600400
TGME49_027970	DNA-binding protein HU, putative	included in other studies - TgHU, Rieff unpublished, PfHU, PMID: 18663012
TGME49_099070	pyruvate kinase, putative	included in other studies - PfPyKII, PMID: 19015045
TGME49_021320	acetyl-CoA carboxylase, putative	included in other studies - ACC1, PMID: 10557330
TGME49_055680	YbaK / prolyl-tRNA synthetases associated domain containing protein	included in other studies - YgbB, Nair et al; in press
TGME49_072290	pyruvate dehydrogenase E1 beta subunit, putative	included in other studies - PDH E1 beta, PMID: 17449654, PMID: 15612915
TGME49_059260	cell division protein, putative	included in other studies - FtsH, PMID: 19450729, PMID: 17822404
TGME49_066760	isocitrate dehydrogenase, putative	included in other studies - ICDH2, PMID: 17784785
TGME49_105980	dihydrolipoyl dehydrogenase protein, putative	included in other studies - PDH E3, PMID: 17449654
TGME49_021330	DNA gyrase subunit A, putative	among 57 tagged
TGME49_047930	syntaxin, putative	among 57 tagged
TGME49_090670	cytosol aminopeptidase	among 57 tagged
TGME49_073730	hypothetical protein	among 57 tagged
TGME49_111690	hypothetical protein	among 57 tagged
TGME49_118510	N-ethylmaleimide-sensitive factor	among 57 tagged
TGME49_014500	protamine P1, putative	among 57 tagged
TGME49_068010	protein kinase, PfEST homolog	among 57 tagged
TGME49_105800	hypothetical protein, conserved	among 57 tagged
TGME49_036530	hypothetical protein	among 57 tagged
TGME49_067050	hypothetical protein	among 57 tagged

369 hits from expression profile based search

TGME49_024570	hypothetical protein	among 57 tagged
TGME49_008840	ATP-dependent DNA helicase, putative	among 57 tagged
TGME49_017690	hypothetical protein	among 57 tagged
TGME49_007090	hypothetical protein, conserved	among 57 tagged
TGME49_015420	SNARE protein, putative	among 57 tagged
TGME49_049310	hypothetical protein	among 57 tagged
TGME49_007100	hypothetical protein	among 57 tagged
TGME49_001270	hypothetical protein	among 57 tagged
TGME49_113640	hypothetical protein	among 57 tagged
TGME49_049690	hypothetical protein	among 57 tagged
TGME49_046770	hypothetical protein	among 57 tagged
TGME49_061680	hypothetical protein	among 57 tagged
TGME49_021920	hypothetical protein	among 57 tagged
TGME49_078160	hypothetical protein	among 57 tagged
TGME49_090600	succinyl-CoA ligase alpha subunit, putative	among 57 tagged
TGME49_032440	hypothetical protein	among 57 tagged
TGME49_070070	vesicle trafficking protein, putative	among 57 tagged
TGME49_091810	hypothetical protein	among 57 tagged
TGME49_087270	hypothetical protein	among 57 tagged
TGME49_095460	got1-like family domain-containing protein	among 57 tagged
TGME49_110770	hypothetical protein, conserved	among 57 tagged
TGME49_085510	hypothetical protein	among 57 tagged
TGME49_054520	hypothetical protein	among 57 tagged
TGME49_020600	hypothetical protein, conserved	among 57 tagged
TGME49_039680	hypothetical protein, conserved	among 57 tagged
TGME49_014560	hypothetical protein	among 57 tagged
TGME49_090030	hypothetical protein, conserved	among 57 tagged
TGME49_082150	hypothetical protein	among 57 tagged
TGME49_016630	hypothetical protein, conserved	among 57 tagged
TGME49_091670	RNA helicase, putative	among 57 tagged
TGME49_033340	hypothetical protein	among 57 tagged
TGME49_016790	ABC transporter, putative	among 57 tagged
TGME49_094250	hypothetical protein	among 57 tagged
TGME49_118150	major facilitator superfamily domain-containing protein	among 57 tagged
TGME49_024150	hypothetical protein	among 57 tagged
TGME49_063510	gamma-tubulin complex component 2, putative	among 57 tagged
TGME49_039320	hypothetical protein, conserved	among 57 tagged
TGME49_068830	hypothetical protein, conserved	among 57 tagged
TGME49_047410	hypothetical protein	among 57 tagged
TGME49_084620	hypothetical protein	among 57 tagged
TGME49_002440	hypothetical protein	among 57 tagged
TGME49_059230	hypothetical protein	among 57 tagged
TGME49_059520	hypothetical protein	among 57 tagged
TGME49_078170	hypothetical protein	among 57 tagged
TGME49_046470	hypothetical protein, conserved	among 57 tagged
TGME49_090860	amino acid transporter, putative	among 57 tagged
TGME49_012940	hypothetical protein	border-line candidate
TGME49_040280	hypothetical protein	border-line candidate
TGME49_022180	hypothetical protein	border-line candidate
TGME49_111290	protein tyrosine phosphatase-like domian-containing protein	border-line candidate
TGME49_057990	heat shock protein, putative	border-line candidate
TGME49_088570	hypothetical protein	border-line candidate
TGME49_021170	peptidase family M48 domain-containing protein	border-line candidate
TGME49_097800	structural maintenance of chromosomes protein, putative	border-line candidate
TGME49_085660	DEAD/DEAH box helicase domain-containing protein	border-line candidate
TGME49_022220	articulin 4	border-line candidate
TGME49_025050	adenosylhomocysteinase, putative	border-line candidate
TGME49_066620	thioredoxin, putative	border-line candidate
TGME49_057450	SCO1/SenC domain-containing protein	border-line candidate
TGME49_121430	DNA repair protein recA , putative	border-line candidate
TGME49_062100	TPR domain-containing protein	border-line candidate
TGME49_018920	proteasome subunit beta type 5, putative	border-line candidate
TGME49_020250	chloride channel, nucleotide-sensitive, 1A, putative	border-line candidate
TGME49_073500	signal transduction protein, putative	border-line candidate
TGME49_049590	proteasome subunit alpha type 5, putative	border-line candidate
TGME49_118310	transketolase, putative	border-line candidate
TGME49_032600	patatin-like phospholipase domain-containing protein	rejected
TGME49_010780	ubiquitin carboxyl-terminal hydrolase, putative	rejected
TGME49_121660	hypothetical protein	rejected

369 hits from expression profile based search

TGME49_072910	TCP-1/cpn60 family chaperonin, putative	rejected
TGME49_058060	hypothetical protein, conserved	rejected
TGME49_031170	chromosome condensation protein, putative	rejected
TGME49_057540	hypothetical protein	rejected
TGME49_019870	DNA replication licensing factor, putative	rejected
TGME49_071200	hypothetical protein	rejected
TGME49_120110	proliferating cell nuclear antigen, putative	rejected
TGME49_094640	ribonucleoside-diphosphate reductase, large subunit, putative	rejected
TGME49_097080	hypothetical protein	rejected
TGME49_059250	ATP-dependent DNA helicase, putative	rejected
TGME49_018780	phosphoserine aminotransferase, putative	rejected
TGME49_008560	mitochondrial carrier domain-containing protein	rejected
TGME49_034190	glycine hydroxymethyltransferase, putative	rejected
TGME49_062560	hypothetical protein	rejected
TGME49_086760	hypothetical protein	rejected
TGME49_020450	ribonuclease H, putative	rejected
TGME49_090150	hypothetical protein	rejected
TGME49_116300	DEAD/DEAH box helicase, putative	rejected
TGME49_069130	hypothetical protein	rejected
TGME49_115110	prefoldin subunit 5, putative	rejected
TGME49_083700	phosphatidylinositol 3- and 4-kinase domain-containing protein	rejected
TGME49_061950	ATP synthase beta chain, putative	rejected
TGME49_009770	DNA repair helicase, putative	rejected
TGME49_022030	hypothetical protein	rejected
TGME49_071860	tRNA (Uracil-5-)-methyltransferase domain-containing protein	rejected
TGME49_077220	hypothetical protein	rejected
TGME49_036800	hypothetical protein	rejected
TGME49_057440	hypothetical protein	rejected
TGME49_073520	proteasome PCI domain-containing protein	rejected
TGME49_028180	cytochrome C oxidase assembly factor COX15, putative	rejected
TGME49_019100	cyclin-dependent kinase, putative	rejected
TGME49_088540	hypothetical protein	rejected
TGME49_043390	hypothetical protein	rejected
TGME49_120570	G1 to S phase transition protein, putative	rejected
TGME49_057730	methionine aminopeptidase, putative	rejected
TGME49_040490	hypothetical protein	rejected
TGME49_116520	1,4-alpha-glucan branching enzyme, putative	rejected
TGME49_044550	hypothetical protein	rejected
TGME49_053540	hypothetical protein	rejected
TGME49_057740	UMP-CMP kinase, putative	rejected
TGME49_071240	KH domain-containing protein	rejected
TGME49_050010	hypothetical protein, conserved	rejected
TGME49_090160	sortilin, putative	rejected
TGME49_011060	hypothetical protein	rejected
TGME49_060410	hypothetical protein	rejected
TGME49_017740	oxoacyl-ACP reductase, putative	rejected
TGME49_026890	hypothetical protein	rejected
TGME49_044300	hypothetical protein	rejected
TGME49_048530	phosphatidylinositol 3- and 4-kinase domain-containing protein	rejected
TGME49_034500	phenylalanyl tRNA synthetase isoform, putative	rejected
TGME49_031910	ATP synthase gama chain, putative	rejected
TGME49_037140	ethylene inducible protein, putative	rejected
TGME49_084010	DNA polymerase I, putative	rejected
TGME49_037220	DNA replication licensing factor, putative	rejected
TGME49_072160	hypothetical protein	rejected
TGME49_005110	hypothetical protein	rejected
TGME49_060680	DNA primase small subunit, putative	rejected
TGME49_049510	hypothetical protein	rejected
TGME49_110360	hypothetical protein, conserved	rejected
TGME49_014310	hypothetical protein	rejected
TGME49_053160	hypothetical protein	rejected
TGME49_077680	hypothetical protein	rejected
TGME49_083710	longevity-assurance (LAG1) domain-containing protein	rejected
TGME49_081480	WD domain, G-beta repeat-containing protein	rejected
TGME49_115530	hypothetical protein	rejected
TGME49_027340	hypothetical protein	rejected

369 hits from expression profile based search

TGME49_051730	hypothetical protein	rejected
TGME49_090620	hypothetical protein	rejected
TGME49_040520	hypothetical protein	rejected
TGME49_006700	hypothetical protein	rejected
TGME49_051800	hypothetical protein	rejected
TGME49_089360	hypothetical protein	rejected
TGME49_073330	hypothetical protein	rejected
TGME49_035440	hypothetical protein	rejected
TGME49_051520	hypothetical protein	rejected
TGME49_095060	hypothetical protein	rejected
TGME49_043920	DNA replication licensing factor, putative	rejected
TGME49_061620	hypothetical protein	rejected
TGME49_100270	hypothetical protein	rejected
TGME49_073850	hypothetical protein	rejected
TGME49_089000	hypothetical protein	rejected
TGME49_011730	SET domain-containing protein	rejected
TGME49_100260	threonyl-tRNA synthetase, putative	rejected
TGME49_015100	hypothetical protein	rejected
TGME49_044690	hypothetical protein	rejected
TGME49_095970	hypothetical protein	rejected
TGME49_003710	hypothetical protein	rejected
TGME49_007060	putative	rejected
TGME49_073800	hypothetical protein, conserved	rejected
TGME49_042810	hypothetical protein	rejected
TGME49_059550	hydroxymethyldihydropterin pyrophosphokinase-dihydropteroate synthase	rejected
TGME49_014970	DNA replication licensing factor, putative	rejected
TGME49_113750	hypothetical protein, conserved	rejected
TGME49_026960	phosphofructokinase, putative	rejected
TGME49_022270	hypothetical protein	rejected
TGME49_017730	hypothetical protein	rejected
TGME49_001250	chypothetical protein, conserved	rejected
TGME49_070360	hypothetical protein	rejected
TGME49_001870	TPR domain-containing protein	rejected
TGME49_086140	hypothetical protein	rejected
TGME49_011070	hypothetical protein	rejected
TGME49_087480	hypothetical protein	rejected
TGME49_082090	hypothetical protein	rejected
TGME49_019080	hypothetical protein	rejected
TGME49_039270	hypothetical protein	rejected
TGME49_062910	NADH-cytochrome B5 reductase, putative	rejected
TGME49_058380	hypothetical protein	rejected
TGME49_044510	hypothetical protein	rejected
TGME49_054820	hypothetical protein	rejected
TGME49_106970	thymidylate kinase, putative	rejected
TGME49_032240	hypothetical protein, conserved	rejected
TGME49_028770	hypothetical protein, conserved	rejected
TGME49_049940	hypothetical protein	rejected
TGME49_089070	P-Type cation-transporting ATPase, putative	NA
TGME49_092080	leucyl-tRNA synthetase, putative	NA
TGME49_051590	protease, putative	NA
TGME49_068850	enolase 2	NA
TGME49_024900	adenylate kinase, putative	NA
TGME49_033140	putative	NA
TGME49_054580	UDP-galactose transporter protein, putative	NA
TGME49_048400	lactoylglutathione lyase, putative	NA
TGME49_032310	endonuclease/exonuclease/phosphatase domain-containing protein	NA
TGME49_040430	glyoxalase, putative	NA
TGME49_049530	exportin, putative	NA
TGME49_057770	SET domain-containing protein	NA
TGME49_043510	OTU-like cysteine protease domain-containing protein	NA
TGME49_089300	methionyl-tRNA synthetase, putative	NA
TGME49_057960	mannose-1-phosphate guanylyltransferase, putative	NA
TGME49_018810	histidyl tRNA synthetase 2	NA
TGME49_052290	importin alpha, putative	NA
TGME49_017910	DNA polymerase alpha catalytic subunit	NA
TGME49_099200	trafficking protein particle complex subunit 3, putative	NA
TGME49_060420	HEC/Ndc80p family protein, putative	NA
TGME49_038050	protein	NA

369 hits from expression profile based search

TGME49_038100	emp24/gp25L/p24 family domain-containing, transmembrane protein, putative	NA
TGME49_019540	alanyl-tRNA synthetase, putative	NA
TGME49_040650	coatomer alpha subunit, putative	NA
TGME49_047510	fructose-1,6-bisphosphatase, putative	NA
TGME49_025310	ARF1-directed GTPase-activating protein, putative	NA
TGME49_094620	putative	NA
TGME49_057490	prefoldin subunit 3, putative	NA
TGME49_066450	lysine decarboxylase domain-containing protein	NA
TGME49_061460	structure specific recognition protein I, putative	NA
TGME49_037890	CAM kinase, CDPK family	NA
TGME49_121590	hypothetical protein	NA
TGME49_030060	acetyltransferase domain-containing protein	NA
TGME49_078280	WD-40 repeat-containing protein	NA
TGME49_051880	tyrosyl-tRNA synthetase, putative	NA
TGME49_014780	putative	NA
TGME49_054110	tryptophanyl-tRNA synthetase, putative	NA
TGME49_002370	TCP-1/cpn60 family chaperonin, putative	NA
TGME49_047610	snRNP protein Lsm5, putative	NA
TGME49_118720	proline synthetase co-transcribed protein, putative	NA
TGME49_037110	replication factor C small subunit, putative	NA
TGME49_031240	chromatin assembly factor 1 subunit, putative	NA
TGME49_043950	prohibitin, putative	NA
TGME49_080690	DNA polymerase epsilon subunit, putative	NA
TGME49_104710	eukaryotic peptide chain release factor subunit, putative	NA
TGME49_076120	zinc finger MYND domain-containing protein	NA
TGME49_036130	Signal recognition particle 9 kDa protein, putative	NA
TGME49_106930	proteasome subunit beta type 7, putative	NA
TGME49_039490	mitochondrial branched-chain alpha-keto acid dehydrogenase E1, putative	NA
TGME49_111080	cation channel family domain-containing protein	NA
TGME49_025240	50s ribosomal protein L13, putative	NA
TGME49_039520	serine:pyruvate/alanine:glyoxylate aminotransferase, putative	NA
TGME49_052310	DNA polymerase epsilon p17 subunit, putative	NA
TGME49_054630	CMGC kinase	NA
TGME49_024610	IQ calmodulin-binding motif domain-containing protein	NA
TGME49_010960	replication factor C subunit, putative	NA
TGME49_002530	aspartyl-tRNA synthetase, putative	NA
TGME49_111500	ubiquitin-activating enzyme, putative	NA
TGME49_050880	adenosine kinase	NA
TGME49_003170	protein	NA
TGME49_034440	aminotransferase, putative	NA
TGME49_027020	NAD-dependent deacetylase, putative	NA
TGME49_037470	aminomethyltransferase, mitochondrial, putative	NA
TGME49_069250	26S proteasome regulatory subunit, putative	NA
TGME49_090010	proteasome subunit beta type 1, putative	NA
TGME49_077070	SNF2 family N-terminal domain-containing protein	NA
TGME49_059950	carbonic anhydrase domain containing protein	NA
TGME49_118730	UDP-N-acetyl-D-galactosamine:polypeptide N-acetylgalactosaminyltransferase T3, putative	NA
TGME49_106080	DEAD/DEAH box helicase, putative	NA
TGME49_112230	DNA topoisomerase II, putative	NA
TGME49_111620	WD repeat domain-containing protein	NA
TGME49_118410	TCP-1/cpn60 family chaperonin, putative	NA
TGME49_031940	thiF family domain-containing protein	NA
TGME49_087500	TCP-1/cpn60 family chaperonin, putative	NA
TGME49_018870	TBC domain-containing protein	NA
TGME49_029990	TCP-1/cpn60 family chaperonin, putative	NA
TGME49_118600	DNA/pantothenate metabolism flavoprotein domain-containing protein	NA
TGME49_014290	intracellular protease, putative	NA
TGME49_039530	alanine--glyoxylate aminotransferase, putative	NA
TGME49_002920	para-aminobenzoate synthase, putative	NA
TGME49_053070	hydrolase, TatD family domain-containing protein	NA
TGME49_005720	adenosine/AMP deaminase domain containing protein	NA
TGME49_095730	TPR domain-containing protein	NA
TGME49_120620	queuine tRNA-ribosyltransferase, putative	NA
TGME49_031350	glucosamine--fructose-6-phosphate aminotransferase (isomerizing), putative	NA

369 hits from expression profile based search

TGME49_043710	TCP-1/cpn60 family chaperonin, putative	NA
TGME49_071250	KH domain-containing protein	NA
TGME49_026000	ATP synthase, putative	NA
TGME49_090640	DNA mismatch repair protein, putative	NA
TGME49_000430	deoxycytidylate deaminase, putative	NA
TGME49_061790	hypothetical protein	NA
TGME49_028300	hypothetical protein	NA
TGME49_053020	hypothetical protein	NA
TGME49_081510	ribonuclease HI large subunit, putative	NA
TGME49_056990	glycyl-tRNA synthetase, putative	NA
TGME49_071810	inhibitor-1 of protein phosphatase type 2A	NA
TGME49_048880	Ras family domain-containing protein	NA
TGME49_024910	CutA1 divalent ion tolerance domain-containing protein	NA
TGME49_039500	proteasome subunit alpha type 4, subunit	NA
TGME49_014580	protein antigen, putative	NA
TGME49_013000	activator 1 36 kDa, putative	NA
TGME49_070900	ATPase, AAA family domain-containing protein	NA
TGME49_058130	Ras family domain-containing protein	NA
TGME49_057030	hypothetical protein	NA
TGME49_063990	hypothetical protein	NA
TGME49_075800	microneme protein, putative	NA
TGME49_022860	putative	NA
TGME49_008390	capping protein alpha-like subunit, putative	NA
TGME49_087210	proteasome subunit alpha type 2, putative	NA
TGME49_048850	methionine aminopeptidase, putative	NA
TGME49_036810	tRNA-Leu	NA
TGME49_061730	hypothetical protein	NA
TGME49_076910	endoplasmic reticulum retention receptor, putative	NA
TGME49_022010	ubiquitin-like domain-containing CTD phosphatase 1, putative	NA
TGME49_009120	hypothetical protein	NA
TGME49_035150	transporter, putative	NA
TGME49_017750	hypothetical protein	NA
TGME49_113080	hypothetical protein	NA
TGME49_068300	hypothetical protein	NA
TGME49_070840	NAD(+) ADP-ribosyltransferase, putative	NA
TGME49_100230	hypothetical protein	NA
TGME49_060150	hypothetical protein	NA
TGME49_115880	hypothetical protein	NA
TGME49_033870	hypothetical protein	NA
TGME49_026720	translin, putative	NA
TGME49_067550	hypothetical protein, conserved	NA
TGME49_067560	protein phosphatases PP1 regulatory subunit, putative	NA
TGME49_080490	ubiquitin conjugation factor, putative	NA
TGME49_088020	hypothetical protein	NA
TGME49_026830	DnaK family protein	NA
TGME49_022160	aldehyde dehydrogenase, putative	NA
TGME49_068340	glycosyltransferase family 28 C-terminal domain-containing protein	NA
TGME49_079360	hypothetical protein	NA
TGME49_062660	hypothetical protein	NA
TGME49_051690	seryl-tRNA synthetase, putative	NA
TGME49_009700	hypothetical protein	NA
TGME49_012730	hypothetical protein	NA
TGME49_085710	hypothetical protein	NA
TGME49_121550	hypothetical protein	NA
TGME49_023530	hypothetical protein, conserved	NA
