# Supplementary material for: A Systematic Screen to Discover and Analyze Apicoplast Proteins Identifies a Conserved and Essential Protein Import Factor
Source: PLoS Pathog. 2011 Dec 1;7(12):e1002392. doi: 10.1371/journal.ppat.1002392 (PMC3228799; doi:10.1371/journal.ppat.1002392)
Supplement: Table S3 — 57 genes chosen to be experimentally addressed, including primers, PCR products sizes and linearization sites. (PDF) [file ppat.1002392.s009.pdf]

# Result of attempt and DNA cloning information for tagging of 57 apicoplast proteins encoding genes

| Gene ID | Predicted product | Result of attempt | LIC F primer | LIC R primer | Size (bp) | Enzyme | RNA seq test comment |
|---|---|---|---|---|---|---|---|
| TGME49_007100 | hypothetical protein | cc | TACTTCCAATCCAATTTAATGCCACCCGGAGAAGACGC | TCCTCCACTTCCAATTTTAGCTTCTATCCGAATACAAGACGC | 809 | EcoRV | |
| TGME49_110770 | hypothetical protein, conserved | Apicoplast | TACTTCCAATCCAATTTAATGCATAGATATCTACATACTCCCATAGAC | | 630 | EcoRV | |
| TGME49_073730 | hypothetical protein | NS | TACTTCCAATCCAATTTAATGCAGTAACATTTATGGGTAG | TCCTCCACTTCCAATTTTAGCGGAGTTCGCATCATTTGCGAG | 1667 | AvrII | pred Ct match RNAseq |
| TGME49_068830 | hypothetical protein, conserved | Mitochondria | TACTTCCAATCCAATTTAATGCTTGAGACAGCCAAGACG | | 1822 | BstBI | |
| TGME49_021920 | hypothetical protein | Apcioplast | TACTTCCAATCCAATTTAATGCATGCTGTGGTTCTTGCGA | TCCTCCACTTCCAATTTTAGCAGGCTCAGGTTCCAATC | 1599 | NsiI | |
| TGME49_021330 | DNA gyrase subunit A, | Apicoplast | TACTTCCAATCCAATTTAATGCTTCGTCCTCGTCTGTGTT | TCCTCCACTTCCAATTTTAGCGTCTTCTGGTGTTCGTCTCTT | 1779 | NcoI | |
| TGME49_014500 | protamine P1, putative | Nucleuse | TACTTCCAATCCAATTTAATGCCACTCGAAGGCGGGTG | TCCTCCACTTCCAATTTTAGCTTCTTCATCTTCGTCATCTTG | 2133 | SnaBI | |
| TGME49_008840 | ATP-dependent DNA helicase, putative | Apicoplast | TACTTCCAATCCAATTTAATGCCGACGGAAGAAAGGGATATCG | TCCTCCACTTCCAATTTTAGCAACTCTAAACAACCAGTCTATCTTCTTG | 2879 | EcoRV | |
| TGME49_090030 | hypothetical protein, conserved | cc | TACTTCCAATCCAATTTAATGCCGTTGAGCGGATGTCTGG | TCCTCCACTTCCAATTTTAGCATGGTGTCCAGCGGCTTCC | 1666 | MluI | pred Ct match RNAseq |
| TGME49_078170 | hypothetical protein | cc | TACTTCCAATCCAATTTAATGCAGGGAGCGCTGCCG | TCCTCCACTTCCAATTTTAGCCCACCCGGCAAGAAAACGC | 852 | PmlI | pred Ct match RNAseq, UTR longer |
| TGME49_078170 | hypothetical protein | cc | | | | | |
| TGME49_087270 | hypothetical protein | Apicoplast | TACTTCCAATCCAATTTAATGCGGCGAGCCTGAAGCTCC | TCCTCCACTTCCAATTTTAGCCATACCAAAGTTTACGCTGAG | 816 | EcoRV | |
| TGME49_049690 | hypothetical protein | Mitochondria | TACTTCCAATCCAATTTAATGCCCCCCGTCCTCGCGTCTG | TCCTCCACTTCCAATTTTAGCGGCTTTTTCAGACGTTTCCTG | 1245 | PstI | |
| TGME49_118150 | major facilitator superfamily domain-containing protein | cc | TACTTCCAATCCAATTTAATGCGGTACATGTGTCGCGCG | TCCTCCACTTCCAATTTTAGCGCTGTGCACTGTGACAGCCAC | 1279 | ApaI | pred Ct match RNAseq, UTR longer |
| TGME49_090860 | amino acid transporter, | NS | TACTTCCAATCCAATTTAATGCGGACCGGGGGCGC | TCCTCCACTTCCAATTTTAGCGACTTCACAAACAGGCTCGA | 1217 | MluI | pred Ct match RNAseq |
| TGME49_016790 | ABC transporter, putative | NS | TACTTCCAATCCAATTTAATGCGAGGTGGCGGCGCCC | TCCTCCACTTCCAATTTTAGCAGCGTCGTCGAGCATGAAAT | 1835 | MluI | pred Ct match RNAseq |
| TGME49_090600 | succinyl-CoA ligase alpha subunit, putative | cc | TACTTCCAATCCAATTTAATGCCACCCCCCTTCGCT | TCCTCCACTTCCAATTTTAGCGTGAAGGCCGGCCGCC | 1464 | XcmI | pred Ct match RNAseq |
| TGME49_090670 | cytosol aminopeptidase | NS | TACTTCCAATCCAATTTAATGCATTCCTCTCTGTCGGGGA | TCCTCCACTTCCAATTTTAGCGTTCTCTTTCGTTTGTGTGCT | 1688 | PmlI | pred Ct match RNAseq |
| TGME49_078160 | hypothetical protein | Cytosol | TACTTCCAATCCAATTTAATGCTCCAACAGCTTTAGAGGCG | TCCTCCACTTCCAATTTTAGCTTCCCGGGCACAGCCAGAAGCTCTACC | 969 | SnaBI | |
| TGME49_047930 | syntaxin, putative | cc | TACTTCCAATCCAATTTAATGCGGGCAGCGGAGAAAGCC | TCCTCCACTTCCAATTTTAGCGGTGTGTTTCATGATCAGCAG | 2466 | PmlI | pred Ct match RNAseq, UTR longer |
| TGME49_015420 | SNARE protein, putative | NS | TACTTCCAATCCAATTTAATGCTGCGGCGTTTCCCAGATG | TCCTCCACTTCCAATTTTAGCGTAAAGCTGACAGCACTGATTGTTTTTC | 1568 | BstBI | pred Ct match RNAseq |
| TGME49_063510 | gamma-tubulin complex component 2, putative | cc | TACTTCCAATCCAATTTAATGCTGCCCCGTCGGGGG | TCCTCCACTTCCAATTTTAGCTCGGCCGCAGCCGGTTCG | 1603 | EcoRV | pred Ct match RNAseq, UTR longer |
| TGME49_118510 | N-ethylmaleimide-sensitive factor | cc | TACTTCCAATCCAATTTAATGCCAGCACCACTTGCAGATCC | TCCTCCACTTCCAATTTTAGCGAAGGGCATTGGGTCATAACTCC | 1939 | SnaBI | pred Ct match RNAseq |
| TGME49_095460 | got1-like family domain-containing protein | cc | TACTTCCAATCCAATTTAATGCCGGTCTGTGCCTCGGAGG | TCCTCCACTTCCAATTTTAGCATACGGCAAGCGGCTTTTGTCC | 353 | ApaI | pred Ct match RNAseq |
| TGME49_070070 | vesicle trafficking protein, putative | See fig | TACTTCCAATCCAATTTAATGCATGCGGCCTCAGGACC | TCCTCCACTTCCAATTTTAGCGAGGAAGAGTTTCCAGAAAATCAAGATC | 1337 | StuI | pred Ct match RNAseq |
| TGME49_111690 | hypothetical protein | NS | TACTTCCAATCCAATTTAATGCATCCCGGGGGGCGAGG | TCCTCCACTTCCAATTTTAGCGCTGAGAAGACGATCAATCG | 766 | BstBI | pred Ct match RNAseq |
| TGME49_007090 | hypothetical protein, conserved | cc | TACTTCCAATCCAATTTAATGCCGTCTTCTGCTTTATCCG | TCCTCCACTTCCAATTTTAGCTTCTATCCGAATACAAGACGC | 1572 | SnaBI | Ct doesn't match |
| TGME49_039680 | hypothetical protein, conserved | Apicoplast | TACTTCCAATCCAATTTAATGCTGAGTCGGCCAAACCTCC | TCCTCCACTTCCAATTTTAGCAAGTTTGATGTTGAGCGGTAC | 1195 | MluI | |
| TGME49_094250 | hypothetical protein | NS | TACTTCCAATCCAATTTAATGCTACTCGCCTACGGGATCC | TCCTCCACTTCCAATTTTAGCTTTTTCCGGAGGATAGAAAC | 2948 | PshAI | Ct doesn't match |
| TGME49_059520 | hypothetical protein | NS | TACTTCCAATCCAATTTAATGCTATGGGATGGATGGAGAG | TCCTCCACTTCCAATTTTAGCGCGAGGCACCAAAGCTGTAA | 1722 | BstBI | pred Ct match RNAseq |
| TGME49_046470 | hypothetical protein, conserved | NS | TACTTCCAATCCAATTTAATGCTGCTGCCGCCTCTCACTGCTGAGCG | TCCTCCACTTCCAATTTTAGCCAGGACGTTCAGGCTGGAGCGCGCG | 1846 | NheI | Ct doesn't match |
| TGME49_068010 | protein kinase, PfEST homolog | Nucleus | TACTTCCAATCCAATTTAATGCGATTTCAGCCTCGCAGAA | TCCTCCACTTCCAATTTTAGCTGCGGGACTTCTGCTTCCCTT | 1216 | PstI | |
| TGME49_017690 | hypothetical protein | Golgi | TACTTCCAATCCAATTTAATGCGAAAGTCCGGGAGAACA | TCCTCCACTTCCAATTTTAGCTCTGCCTCCCCATCTTTCTCC | 1337 | BglII/MluI | |
| TGME49_091670 | RNA helicase, putative | Apicoplast | TACTTCCAATCCAATTTAATGCGTTGGGAAACTTCGGTGT | TCCTCCACTTCCAATTTTAGCATTTTTAGGCAGTTCGTCTTC | 1370 | EcoRV | |
| TGME49_014560 | hypothetical protein | Nucleus | TACTTCCAATCCAATTTAATGCGCACCGGCGCCGGTTCTG | TCCTCCACTTCCAATTTTAGCGGATGGTGTTTCCGCAGATCC | 1083 | NruI/BstBI | |
| TGME49_061680 | hypothetical protein | Nucleus | TACTTCCAATCCAATTTAATGCTTCTTCAGGCCCCGCAAGAGCCGCC | TCCTCCACTTCCAATTTTAGCGGCCTCAAGTCCCCGAGTGAAGTGC | 1956 | PstI | |
| TGME49_105800 | hypothetical protein, conserved | Vesicles | TACTTCCAATCCAATTTAATGCGGCTCTGCTGTTGGTTGG | TCCTCCACTTCCAATTTTAGCAACCTCTCTCCGAAAAGATG | 984 | XcmI | pred Ct match RNAseq |
| TGME49_113640 | hypothetical protein | NS | TACTTCCAATCCAATTTAATGCCCTGTTTTCCCTGGCCGT | TCCTCCACTTCCAATTTTAGCGGAGCCACCTGTTTCTTTGTT | 584 | SnaBI | pred Ct match RNAseq |
| TGME49_036530 | hypothetical protein | NS | TACTTCCAATCCAATTTAATGCACGGACCGGGCGGGGGC | TCCTCCACTTCCAATTTTAGCCGACTGTGCGCTCCCGGCCT | 925 | NruI | Ct doesn't match |
| TGME49_067050 | hypothetical protein | Cytosol | TACTTCCAATCCAATTTAATGCTCGTTCTGCTGCTGTAAGCGC | TCCTCCACTTCCAATTTTAGCGGCCTTCTTGCAGCTGAGGCCGTTTTCG | 1235 | NruI | |
| TGME49_049310 | hypothetical protein | NS | TACTTCCAATCCAATTTAATGCTTTTGAGACGGGGAAC | TCCTCCACTTCCAATTTTAGCCTCAGAAAGTCCCGCGAGCC | 1383 | PstI | pred Ct match RNAseq, UTR longer |
| TGME49_001270 | hypothetical protein | Apicoplast | TACTTCCAATCCAATTTAATGCGCGAGGTGGTTCGTTAGGTGGGGG | TCCTCCACTTCCAATTTTAGCACCAAACAACTGATTATCTTTCAGTCTCTG | 1574 | StuI | |
| TGME49_046770 | hypothetical protein | Nucleus | TACTTCCAATCCAATTTAATGCGACGAGTGTCCCGCAAC | TCCTCCACTTCCAATTTTAGCCGCAGGCGGTTTAGACGGGC | 1692 | BstBI | |

CC - Cannot be Cloned

NS - No Signal detected in IFA with stabely transfected parasites

# Result of attempt and DNA cloning information for tagging of 57 apicoplast proteins encoding genes

| | | | | | | | |
|---|---|---|---|---|---|---|---|
| TGME49_032440 | hypothetical protein | ER | TACTTCCAATCCAATTTAATGCATGGCGCTCTTGCCGAAG | TCCTCCACTTCCAATTTTAGCCTCCTCCGTGTCTTCTGCGTC | 918 | SnaBI | pred Ct match RNAseq |
| TGME49_091810 | hypothetical protein | See fig | TACTTCCAATCCAATTTAATGCGAGTGTACGTACGCAGGC | TCCTCCACTTCCAATTTTAGCATTGTTGTCCGACTCGTCATC | 1931 | XcmI | |
| TGME49_085510 | hypothetical protein | NS | TACTTCCAATCCAATTTAATGCAGCCAGACTTCGTTGTGA | TCCTCCACTTCCAATTTTAGCGGCTCCCGGATTCGGGCGCA | 1187 | MluI | pred Ct match RNAseq |
| TGME49_054520 | hypothetical protein | NS | TACTTCCAATCCAATTTAATGCGTTCGGCGTTACTGTGCT | TCCTCCACTTCCAATTTTAGCAGAGCCTGGCGCCGAGTCTG | 493 | PshAI | pred Ct match RNAseq, UTR longer |
| TGME49_020600 | hypothetical protein, conserved | Vesicules | TACTTCCAATCCAATTTAATGCAGCTGCACCATCCCTCGT | TCCTCCACTTCCAATTTTAGCGGCCATCTCGACAGCATGCG | 1991 | NheI | pred Ct match RNAseq |
| TGME49_033340 | hypothetical protein | ER | TACTTCCAATCCAATTTAATGCGGACAACCTGGGTCGGC | TCCTCCACTTCCAATTTTAGCCTTACTTTTCACACTCCGATC | 897 | PstI/MluI | pred Ct match RNAseq, UTR longer |
| TGME49_024150 | hypothetical protein | Golgi | TACTTCCAATCCAATTTAATGCAACAGAGGGCGGCGCGTTCCTCG | TCCTCCACTTCCAATTTTAGCCACATCGAGAAGCAGAGCGACATCTCC | 1040 | NruI | |
| TGME49_039320 | hypothetical protein, conserved | Apicoplast | TACTTCCAATCCAATTTAATGCAACAGAAGGGTGTATGGACCTGGCG | TCCTCCACTTCCAATTTTAGCGTCTGAGGCCTCTGCATGAATAGCC | 1281 | MluI | |
| TGME49_047410 | hypothetical protein | NS | TACTTCCAATCCAATTTAATGCTGCGTGTATTTTCTTTGGG | TCCTCCACTTCCAATTTTAGCCACTCGGTACCGATCTTGCCT | 1418 | PstI | pred Ct match RNAseq, UTR longer |
| TGME49_084620 | hypothetical protein | NS | TACTTCCAATCCAATTTAATGCAAGTACACCTGGCTGAA | TCCTCCACTTCCAATTTTAGCGTCGCTCTTTTCGGACCTTTC | 1539 | StuI | Ct doesn't match |
| TGME49_002440 | hypothetical protein | Apicoplast | TACTTCCAATCCAATTTAATGCCTCTGGACCGAATCCGGAGAAGACC | TCCTCCACTTCCAATTTTAGCCTGGGGTTTCTCAGTGTCGCGTTCACG | 1485 | PstI | |
| TGME49_016630 | hypothetical protein, conserved | Vesicles | TACTTCCAATCCAATTTAATGCTGCAGATCCCTGATGGAGGGTTGCC | TCCTCCACTTCCAATTTTAGCCTCGTCTTCCGGTTTGAGGGGAGGTGG | 1642 | MluI | pred Ct match RNAseq, UTR longer |
| TGME49_059230 | hypothetical protein | Apicoplast | TACTTCCAATCCAATTTAATGCGCTCGCAGACTTGCCTGGCCTG | TCCTCCACTTCCAATTTTAGCGACATTGAAGTTCTTCAGGCCTTCTTTG | 1276 | MluI | |
| TGME49_024570 | hypothetical protein | NS | TACTTCCAATCCAATTTAATGCCGCTGCACAGGCGC | TCCTCCACTTCCAATTTTAGCAGAAACAGTCAGGAAGCTGAAAAGTTC | 931 | BmgBI | pred Ct match RNAseq, UTR longer |
| TGME49_082150 | hypothetical protein | Apicoplast | TACTTCCAATCCAATTTAATGCCAGGGCGTCCAGCCGCCGGTCCC | TCCTCCACTTCCAATTTTAGCTCCCACAGCCTCTTGCACGTC | 1250 | XcmI | |

CC - Cannot be Cloned

NS - No Signal detected in IFA with stabely transfected parasites
