# Supplementary material for: A Systematic Screen to Discover and Analyze Apicoplast Proteins Identifies a Conserved and Essential Protein Import Factor
Source: PLoS Pathog. 2011 Dec 1;7(12):e1002392. doi: 10.1371/journal.ppat.1002392 (PMC3228799; doi:10.1371/journal.ppat.1002392)
Supplement: Table S4 — Other primers used in this study. (PDF) [file ppat.1002392.s010.pdf]

Other primers used in this study (not included in TableS3)

Primer number	Sequence	Restirction enzyme	Description	Purpose
1525	cggtgattaattacgccccgccctgcc		Reverse CAT	
1524	ggcagggcggggcgtaattaatcaccg		Forward CAT	mutate PacI site in CAT ORF in pLIC_CAT
2156	cccgatatcataatcatgcgtgtagcttcattgtccg	EcoRV	forward TpPPP1 pNR	
2158	cccggtaccaatactgagattcaacgacaatcc	KpnI	reverse TpPPP1 pNR	GFP-tag TpPPP1 and express in Tp
1380	caaaatactgcatggtctagccgacttctctttcttattctcctcg caaaaggtacccgtacgacgtcccggactac		forward primer to modify ToxoY50	
1381	ttgtcaaagaccgcctgacggcatttttctctgaagcaatcgttc gttttatacgactcactatagggcgaattgg		reverse primer to modify ToxoY50	cosmid recombineering for KO of TgKu80
1410	ccgggacgtcgtacgggtacc		reverse in cosmid modification cassette	
1408	ccggcggacgcctcgcagtgcg		reverse dowstream of end of TOXOY50 cosmid	
1412	cagggtgcagttgaggagtgtc		forward in Ku80 first intron	
2305	ggtcgagatggcccgacgg		forward for TgKu80 southern probe	
2306	gcgaatgaggaagagagcacctc		reverse for TgKu80 southern probe	confirm integration for TgKu80 KO
G13_CosMod_F	caggagaaaatatgggggatgcttgggggccgcgttgttcga tgccactgaggtacccgtacgacgtcccggactac		forward primer to modify ToxoW30	
G13_CosMod_R	cacggtcgcctttttatttgtgtttcgcattcccttctggcactaca taccatacgactcactatagggcgaattgg		reverse primer to modify ToxoW30	
1808	gcaaacgagggggaaatggaagc		reverse downstream to cosmid ToxoW30 to test integration	cosmid recombineering for KO of PPP1
1683	gggagatctatggcgtctctctgtctccacc	BglII	forward to test potential start sites	
1684	gggagatctatgagttccccttttctgcgcc	BglII	forward to test potential start sites	
1685	gggagatctatgttcacaccctctgttacttctc	BglII	forward to test potential start sites	
1686	gggagatctatgacgggtggaggttcggac	AvrII	forward to test potential start sites	
1687	gggcctaggcataccaaagtttacgctgagcc	AvrII	reverse to clone with Myc tag	test PPP1 gene model and express ectopic copy
1756	ccccatatggtacgccttcactcacggtc	NdeI	reverse to amplify PPP1 Pre-promoter region	
1759	gggcatatgcgctatcctggcttttactgtgg	NdeI	forward to amplify PPP1 Pre-promoter region	PPP1 promoter replacement
1154	cgcacggcagtcagataacaggtgta		reverse in DHFR cassette	
1592	cgccttggcgaatgttcatgac		forward on T7S4 promoter	confirm integration for promoter replacement
2304	cctcgattcctctctccgggc		forward for PPP1 southern probe	southern blot probe
1913	gggagatctatggcactccggatcgcggagg	BglII	forward to test potential start sites	
1914	gggagatctatgggagtgctcgtgcttgc	BglII	forward to test potential start sites	
1915	gggagatctatgctttctgtgaccccgc	BglII	forward to test potential start sites	
1916	gggcctaggaagtttgatgttgagcggtactgg	AvrII	reverse to clone with Myc tag	test TGME49_039680 gene model and express ectopic copy
2088	gggattaataggtgactccgcctcctttttctcg	MseI	TGME49_039680_PR_F	
2089	cccattaatttcattgttccgttttcagaccgtg	MseI	TGME49_039680_PR_R	TGME49_039680 promoter replacement
2293	ggcacaacgaagttccgggg		reverse to test PR	confirm integration for TGME49_039680 promoter replacement
2292	ggcagctggagagaaggaggcaaggcc		forward to test PR	
1382	tacttccaatccaatttaatgcgagactatgtaacgcagacg		forward ACP in LIC	
1383	tcctccacttccaattttagccgctgtagcgctcctgagtc		reverse ACP in LIC	ACP tagging endogenously
