# Supplementary material for: A Systematic Screen to Discover and Analyze Apicoplast Proteins Identifies a Conserved and Essential Protein Import Factor
Source: PLoS Pathog. 2011 Dec 1;7(12):e1002392. doi: 10.1371/journal.ppat.1002392 (PMC3228799; doi:10.1371/journal.ppat.1002392)
Supplement: Table S5 — Accession numbers for genes used in alignments or phylogeny. (PDF) [file ppat.1002392.s011.pdf]

Accession numbers and domain prediction e-values for PPP1 and ATrx2 homologs

BLAST results for PPP1

Organism	Accession number
Nucleotide databases	
Apicomplexa	
Toxoplasma gondii	TGME49_087270 (ToxoDB)
Plasmodium falciparum 3D7	PFL0600w
Plasmodium vivax	PVX_084635
Plasmodium knowlesi	PKH_131100
Plasmodium chabaudi	PCAS_061110
Plasmodium yoelii PY06936	PY06936
Plasmodium berghei	PB001041.00.0
Neospora caninum	FP207527.1
Others chromalveolata	
Thalassiosira pseudonana	XM_002293706.1
Phaeodactylum tricornutum	XM_002183343.1
Guillardia theta nucleomorph chromosome 1	XM.001713580.1
Hemiselmis andersenii nucleomorph Chromosome 3	XM_001712543.1
Cyanidioschyzon merolae chromosome 9	AP006491.2
Fragilariopsis cylindrus	protein ID - 181841 (JGI)
ESTs	
Porphyra yezoensis	AV431972.1
emiliana huxley	FP207527.1, FP232544.1, CX775553.1
Ectocarpus siliculosus	FP296628.1, FP271190.1
Chromera velia	HO866139.1
Aureococcus anophagefferens	FC010487.1
Pseudochattonella farcimen	FR735641.1
Galdieria sulphuraria	(http://genomics.msu.edu/galdieria/blast/blast.cgi)

BLAST results for ATrx2

Organism	Accession	BALST e-value	Predicted Trx location	Superfamily prediction e-value
Toxoplasma gondii	XM_002364282.1	0	173-244	4.05E-03
Plasmodium falciparum	AL844504.1	7.00E-15	81-167	6.26E-07
Plasmodium yoelii yoeli	XM_721231.1	3.00E-17	74-134	3.69E-08
Plasmodium knowlesi	XM_002259484.1	5.00E-21	84-142	1.52E-06
Plasmodium vivax	XM_001613617.1	5.00E-20	28-85	4.08E-07
Plasmodium berghei	XM_670898.1	7.00E-19	74-134	4.36E-08
Hemiselmis andersenii	XM_001712533.1	2.00E-22	4-90	7.84E-07
Cyanidioschyzon merolae	AP006500.2	2.00E-18	8-92	4.37E-07
Guillardia theta	XM_001713216.1	3.00E-21	9-90	9.13E-07
Cryptomonas paramecium	CP002174.1	2.00E-17	9-91	8.73E-05
Desulfobacca acetoxidans	CP002629.1	2.00E-13	8-92	4.37E-07
Prymnesium parvum	DV097556.1	1.00E-19	28-115	6.60E-05
Emilia huxleyi	FP207693.1	2.00E-16	36-116	7.53E-03
Cyanophora paradoxa	EC660113.1	6.00E-15	21-108	2.58E-06
Ectocarpus siliculosus	FP260169.1	1.00E-14	92-175	2.61E-05
Babesia bovis	XM_001609972.1	3.00E-11		none
Neospora caninum	FR823392.1	1.00E-18		none
Grateloupia lanceola	HM766988.1	1.00E-15		none
Theileria parva	XM_758582.1	1.50E-01		none
Plasmodium chaboudi	XM_729255.1	0.23	73-109	0.000342
